# EP4 Antagonism by E7046 diminishes Myeloid immunosuppression and synergizes with Treg-reducing IL-2-Diphtheria toxin fusion protein in restoring anti-tumor immunity

**DOI:** 10.1080/2162402X.2017.1338239

**Published:** 2017-06-28

**Authors:** Diana I. Albu, Zichun Wang, Kuan-Chun Huang, Jiayi Wu, Natalie Twine, Sarah Leacu, Christy Ingersoll, Lana Parent, Winnie Lee, Diana Liu, Renee Wright-Michaud, Namita Kumar, Galina Kuznetsov, Qian Chen, Wanjun Zheng, Kenichi Nomoto, Mary Woodall-Jappe, Xingfeng Bao

**Affiliations:** a Andover Innovative Medicines Institute, Eisai Inc., Andover, MA, USA; b Oncology Business Group, Eisai Inc., Woodcliff Lake, NJ, USA

**Keywords:** Anti-tumor immunity, myeloid cells, PGE_2_, EP4 antagonism, T regulatory cells

## Abstract

Reprogramming of immunosuppressive tumor microenvironment (TME) by targeting alternatively activated tumor associated macrophages (M2TAM), myeloid-derived suppressor cells (MDSC), and regulatory T cells (Tregs), represents a promising strategy for developing novel cancer immunotherapy. Prostaglandin E2 (PGE_2_), an arachidonic acid pathway metabolite and mediator of chronic inflammation, has emerged as a powerful immunosuppressor in the TME through engagement with one or more of its 4 receptors (EP1-EP4). We have developed E7046, an orally bioavailable EP4-specific antagonist and show here that E7046 has specific and potent inhibitory activity on PGE_2_-mediated pro-tumor myeloid cell differentiation and activation. E7046 treatment reduced the growth or even rejected established tumors *in vivo* in a manner dependent on both myeloid and CD8^+^ T cells. Furthermore, co-administration of E7046 and E7777, an IL-2-diphtheria toxin fusion protein that preferentially kills Tregs, synergistically disrupted the myeloid and Treg immunosuppressive networks, resulting in effective and durable anti-tumor immune responses in mouse tumor models. In the TME, E7046 and E7777 markedly increased ratios of CD8^+^granzymeB^+^ cytotoxic T cells (CTLs)/live Tregs and of M1-like/M2TAM, and converted a chronic inflammation phenotype into acute inflammation, shown by substantial induction of STAT1/IRF-1 and IFNγ-controlled genes. Notably, E7046 also showed synergistic anti-tumor activity when combined with anti-CTLA-4 antibodies, which have been reported to diminish intratumoral Tregs. Our studies thus reveal a specific myeloid cell differentiation-modifying activity by EP4 blockade and a novel combination of E7046 and E7777 as a means to synergistically mitigate both myeloid and Treg-derived immunosuppression for cancer treatment in preclinical models.

## Abbreviations

BM: bone marrow
CM: complete medium
DCs: dendritic cells
EP4: prostaglandin E2 receptor 4
gMDSC: granulocytic myeloid-derived suppressor cells
mMDSC: monocytic myeloid-derived suppressor cells
MSD: Meso Scale Discovery
PBMC: peripheral blood mononuclear cells
PGE_2_: prostaglandin E2
TCR: T cell receptor
TAM: tumor-associated macrophages
TME: tumor microenvironment
Treg: T regulatory cells

## Introduction

Checkpoint inhibitors have produced impressive therapeutic effects in cancer patients, but thus far, only a fraction of patients benefit from this class of cancer immunotherapies. It is therefore of great interest to develop novel immunotherapies and combinations to increase patient responsiveness. In this regard, reprogramming of the immunosuppressive TME by targeting myeloid-derived suppressor cells (MDSC), M2TAMs, and Tregs represents an important approach to developing novel cancer immunotherapies.

One immunosuppressive mechanism used by various tumors is production of PGE_2_,^1^ a lipid product of the arachidonic acid metabolism cascade and an inhibitory damage-associated molecular pattern (iDAMP) in the TME.^2^ PGE_2_ controls a large variety of biologic functions, through its binding to E-type prostanoid receptors (EP1 to EP4). The relative contribution of each of these EP receptors in mediating the immunosuppressive effect of PGE_2_ has not been fully elucidated and some reports even show opposing activities of EP receptors on immune function.^3^ EP4, a G_αs_ protein coupled receptor expressed mainly on myeloid cells, T lymphocytes, and tumor cells^4^ has emerged as a major contributor to PGE_2_-mediated enhancement of tumor survival pathways and also as a suppressor of innate and adaptive anti-tumor immune responses, by triggering cancer-promoting chronic inflammation.^5^ This chronic inflammation is orchestrated by PGE_2_ through its activity on a variety of immune cells present within the TME including macrophages,^6-8^ DCs,^9^ CD4^+^ T helper cells,^10-12^ CTLs,^13^ and Tregs.^14^ Given the broad nature of PGE_2_ biologic activities and the differential involvement of EP receptors in mediating these activities, specific targeting of EP4 signaling should provide a more focused and possibly safer approach to anti-cancer therapy than inhibitors of the entire pathway leading to PGE_2_ production.

The TME often contains a large number of myeloid cells, mainly MDSCs and TAMs, whose presence is associated with poor prognosis in a substantial proportion of solid tumors.^15^ Both monocytic MDSCs (mMDSCs), defined as CD11b^+^Ly6c^+^Ly6G^−^, and granulocytic (gMDSCs) defined as CD11b^+^Ly6c^−^Ly6G^+^ are found in the TME and peripheral lymphoid tissues of animal models. MDSCs exert immunosuppressive activity mainly through arginase and production of reactive oxygen species. The less numerous but longer-lived mMDSCs have been shown to be more immunosuppressive on a per cell basis in the TME than the more abundant gMDSCs.^16^ While CD11b^+^Ly6c^lo^MHCII^hi^ M1-like macrophages, or classically activated macrophages, are activated by IFN-γ and sterile or infectious injury, and are tumoricidal^17^; alternative activated M2TAMs, defined as CD11b^+^Ly6c^−^MHCII^lo^, support tumor progression and metastasis through remodeling of extracellular matrix and release of factors that promote cell proliferation, angiogenesis and migration.^18^ PGE_2_ has been shown to favor the immunosuppressive M2TAM phenotype,^19^ therefore, blocking PGE_2_ activity in the TME should reduce M2 polarization. Other important mediators of immunosuppression within the TME are Treg cells which normally control key aspects of self-tolerance. In the TME, Tregs contribute to immunosuppression by maintaining immune tolerance to the tumor and opposing cytotoxic T cell activity.^20^ Treg cells express high levels of CD25 and CTLA-4 and are defined by their expression of the transcription factor Foxp3. Administration of denileukin diftitox, an IL-2-diphtheria toxin fusion protein, has been shown to diminish the number of CD25^high^CD4^+^ Treg cells in patients.^21-23^ Similarly, a key anti-tumor property of anti-CTLA-4 antibodies has been attributed to their ability to reduce Tregs as well.^24,25^ We thought to identify an agent or a combination that can simultaneously target both intratumoral myeloid cell differentiation and Treg viability to efficiently restore the anti-tumor immunity.

In this report, we evaluated myeloid cell-modifying effects of EP4 antagonism *in vitro* and *in vivo*, examined pharmacologically the concurrent targeting of EP4 signaling and Treg viability in tumor-bearing immunocompetent mice, and investigated the mechanisms driving the combined anti-tumor activity of these agents through a combination of genetic, molecular and cellular phenotypic analyses. To selectively antagonize PGE_2_-EP4 signaling we used E7046 (also known as ER-886406),^26^ a clinical stage novel and orally bioavailable small molecule EP4 antagonist (NCT02540291). To reduce Tregs we used E7777, a fusion protein comprising human IL-2 and fragments of diphtheria toxin, representing an improved-purity version of denileukin diftitox. We show here a specific myeloid cell differentiation and anti-tumor activity of E7046 by antagonism of EP4 signaling and a highly potent anti-tumor activity of the novel E7046 and E7777 combination in preclinical cancer models by synergistic inhibition of both myeloid and Treg–induced TME immunosuppression.

## Materials and methods

### Mice

Mice with floxed EP4 gene were acquired from the Breyer Lab of Vanderbilt University^27^ and ER-Cre mice were purchased from Taconic. Both parent strains were on the C57BL/6 background and were crossed to create mice that were homozygous for the floxed EP4 gene and heterozygous for the ER-Cre gene. The colony was maintained and breeding performed at Charles River Laboratories (Wilmington, MA). To produce the knockout of EP4, the mice were orally dosed once daily with tamoxifen (Sigma, St. Louis) 1 mg/kg formulated in sunflower oil. Mice were dosed for 5 consecutive days to generate complete knockout. To verify the efficacy of the knockout, blood and tissue samples were collected and analyzed by qPCR for EP4 expression. BALB/c, C57BL/6, and A/J mice were purchased from Jackson Laboratory (Bar Harbor, ME). Nude mice were obtained from Charles River Laboratories (Wilmington, MA). Experiments were conducted with 6–10 week-old mice, unless otherwise specified. All mice were kept under specific pathogen-free conditions in the Life Science Facility of AIM/Eisai Inc. (Andover, MA), and animal procedures were conducted under conditions approved by the Institutional Animal Care and Use Committee following the guidance of the Association for Assessment and Accreditation of Laboratory Animal Care (AAALAC).

### Cell culture, antibodies, agonists, antagonists and other reagents

MDA-MB-231 and COLO-205 human cancer cell lines were obtained from ATCC (American Type Culture Collection, Manassas, VA). HMVEC were obtained from Lonza. Mouse cell lines CT26, LL2, 4T1, EMT6, SAI/N, and were obtained from ATCC, mouse cell line Pan02 was obtained from the DCTD-NCI (Frederick, MD) and mouse cell line H22 from GenScript (Piscataway, NJ). All cell lines were cultured according to vendor recommended growth media with 10–15% FBS and conditions (37°C and 5% CO_2_). Cell lines from early passage stocks (less than 5) were stored at -180°C in the Eisai Biobank. Cell lines were used in assays before reaching 15 passages in culture. Cell line authentication was conducted by the Short Tandem Repeat (STR) profiling method at ATCC.

E7046 was formulated in 0.5% methylcellulose (MC) and administered by oral gavage at 100 mg/kg (H22 model) or 150 mg/kg (all other *in vivo* studies). For *in vitro* studies, E7046 was dissolved in DMSO to form a 30 mM stock solution, which was further diluted for specific experiments. E7777, supplied as a sterile 156 µg/mL stock solution, was diluted in saline for injection. For *in vivo* cell-depletion studies, *in vivo* Plus anti-mouse CD4 (clone GK1.5) and anti-CD8 (clone 53–6.72) antibodies from BioXCell (West Lebanon, NH) were administered i.p. *As controls, in vivo* Plus Rat IgG2b (clone LTF-2) and Rat IgG2a isotype control (clone 2A3) (BioXCell) were injected in the control mice. *In vivo* Plus anti-PD1 (clone RMP1–14), anti-CTLA-4 (clone9H10) antibodies and Syrian hamster IgG control (Bi XCell) were diluted in sterile PBS and injected i.p. PGE_2_, SC-51089, ER-880696, and L-798106 were dissolved in DMSO at 10 mM stock concentration.

### Syngeneic mouse tumor studies

For the LL2 tumor growth in EP4^−/−^ mice, EP4 wild type (WT) n = 10, and EP4^−/−^ n = 9 mice were injected subcutaneously (s.c.) with 1 × 10^5^ cells/mouse in 100 µL sterile HBSS. Tumor growth was monitored by tumor volume measurement every 3–4 d throughout the experiment. Tumor volume was calculated by the formula of a prolate ellipsoid: (Lx2W)/2 where L and W are the respective orthogonal length and width measurements (mm).

For the tumor isograft efficacy studies, 6-week old female BALB/c mice were implanted with cancer cells: 1 × 10^5^ CT26 or 4T1 cells or 8 × 10^5^ H22 cells per mouse s.c., or 1 × 10^5^ EMT6 cells in the mammary fat pad. C57BL/6 mice were implanted s.c. with 1 × 10^6^ Pan02 cells per mouse, and A/J mice were implanted s.c. with 2–3 mm^3^ SAI/N tumor fragments. To investigate the role of T cells in the anti-tumor response, 6 week old female nude mice (which lack T cells) were injected s.c. with 1 × 10^5^ CT26 cells. When tumors reached approximately 50–100 mm^3^, tumor–bearing mice were randomly assigned to vehicle or treatment groups, and treatment regimens began. E7046 was administered per oral (p.o.) as a 100 or 150 mg/kg suspension in 0.5% MC, daily for 21 d (QDx21). For the combination studies, E7777 was administered intravenously (i.v.) at 2.5 µg/mouse in saline, as 2 to 3 doses injected one week apart (Q7Dx2–3). Tumor volumes and body weights were recorded 2–3 times a week. For comparison with current immunotherapies, in addition to vehicle control and E7046 + E7777 groups, mice were assigned to anti-PD1 antibodies or anti-mouse PD-1 + anti-mouse CTLA4 antibodies treatment groups. Anti-PD-1 and anti-CTLA-4 antibodies (1 mg/mL), were administered i.p. in 100 µL, 3 times 4 d apart (Q4Dx3) for a total of 300 µg each. Isotype controls were administered i.p. at 1 mg/mL to the control group. For the CD4^+^T and CD8^+^T lymphocyte depletion, anti-mouse CD4 or anti-mouse CD8 antibodies or their isotype controls were administered in 100 µL, i.p. at 2.5 mg/mL every 4 days, for a total of 4 injections per mouse (1 mg). To deplete macrophages, clodronate-containing or control liposomes (Encapsula Nanosciences) were administered i.p. at 1 mg/mouse, every other day for a total of 7 injections. For the live H22 cell challenge, mice whose initial tumors had completely and stably disappeared were paired with age-matched naïve controls and injected s.c. with 8 × 10^5^ H22 cells, on the opposite side from the original implantation site. Tumor volumes and body weights were measured 2 times weekly throughout all experiments.

### In vitro APC differentiation and suppression of T cell proliferation assays

Bone marrow (BM) cells were flushed from femurs of BALB/c mice using sterile CM. Freshly harvested (BM) cells (0.5 × 10^6^) were differentiated in the presence of 20 ng/mL recombinant mouse GM-CSF (Peprotech), ± PGE_2_ (10 nM), at 37°C, for 8 d. CM + fresh GM-CSF ± PGE_2_ was changed on days 3 and 6. After *in vitro* differentiation, cells were analyzed by flow cytometry. For certain experiments, CT26, 4T1 cell supernatants, and/or EP1 (SC-57089), EP2 (ER-880696), EP3 (L-798106), or EP4 (E7046) antagonists at 1 µM, were added to the BM cells. To assess the effect of differentiated BM cells on T cell proliferation, mouse BM cells differentiated as described above were co-cultured for 72 hours with anti-CD3/CD28 Dynabeads-stimulated and CFSE (1 µM)-stained T cells. T cell proliferation was assessed by CFSE dilution using flow cytometry.

Monocytes were purified from PBMCs isolated from healthy volunteer donors or cancer patients using a monocyte separation kit (Miltenyi), and placed in culture with recombinant human GM-CSF (20 ng/mL) + human IL-4 (500 U/mL) ± PGE_2_ (10 nM) ± E7046 (10, 100, 300, 1000, or 3000 nM) for 7 d. Differentiated myeloid cells were identified by surface marker phenotyping by flow cytometry. For the suppression of T cell proliferation, monocytes differentiated as described above were then co-cultured with previously purified autologous T cells (Miltenyi T cell separation kit) at a 1:4 ratio for 96 h. To assess proliferation, T cells were stained with CFSE at 1 µM (Life Technologies) before co-culture with APCs. T cells were stimulated with antiCD3/CD28 Dynabeads according to the manufacturer's protocol (Life Technologies). T cell proliferation was assessed by CFSE dilution using flow cytometry.

### Cytokine and chemokine measurements

Tumor samples were collected in liquid nitrogen, thawed on ice, then minced and lysed in lysis buffer containing a cocktail of protease inhibitors (Roche). Lysates were incubated for 1 h at 4°C on a rotator, sonicated at 4°C for 5 min with intermittent on/off every 30 sec, and centrifuged (6700 x *g*) for 10 min at 4°C. Protein concentration was determined in the supernatants using the BCA assay (Pierce). Mouse cytokines and chemokines were measured in CT26 tumor lysates on days 3, 7, and 14 of E7046 treatment, using the Proinflammatory Panel 1 (mouse) multiplex assay kit (K15048D) and the Sector Imager 2400 from MSD according to the manufacturer's instructions.

### Flow cytometry

FcR-blocked BM-derived APCs, differentiated human PBMC-isolated monocytes, murine splenocytes, or tumor cells were stained for flow cytometry while on ice for 20 min. To obtain single cell suspensions for analysis, tumors were excised from killed mice, cut into small pieces, and digested in medium containing a mix of 1 mg/mL type IV collagenase, 0.25 mg/mL type I collagenase (Worthington Biochemical Corporation), and 0.02 mg/mL DNAse (Roche). Digestion was performed in a 37°C-heated shaker for 30 min, followed by passage through a 70-µm filter. Surface-staining antibodies were added for 30 min after the FcR blocking antibodies. The following antibodies were obtained from BD Biosciences: anti-mouse CD45 (B6.F11), -CD11b (M1/70), -Ly6G (1A8), anti-human CD86 (2331), and -CD16 (3G8). The following Biolegend-manufactured antibodies were used for cell staining: anti-mouse CD11c (N418), anti-human CD1a (HI149), -CD163 (GH/61), and -CD206 (15–2). The following antibodies were purchased from eBioscience: anti-mouse CD4 (GK1.5), -CD8α (53–6.7), -CD25 (PC61.5), - F4/80 (BM8), -MHCII (M5/114.15.2), -PD-L1 (MIH5) –Ly6c (HK1.4), -Foxp3 (FJK-16s), -granzyme B (NGZB), -Gr1 (RB6–8C5). Zombie NIR fixable viability dye was used to determine cell death (Biolegend). Intracellular staining for granzyme B and Foxp3 was performed after surface staining, fixation, and subsequent permeabilization following the manufacturer's protocol. Flow cytometry acquisition was conducted on FACS Canto instruments (I and II), (Becton Dickinson) and data were analyzed using FlowJo software (Tree Star, Inc.).

### RNA isolation, Affimetrix GeneChip® mouse transcriptome assay, and real-time PCR

Total RNA from tumor tissues was isolated using RNeasy Mini Kit (Qiagen) as per the manufacturer's instructions. RNA concentrations were determined by Nanodrop (ND-1000). Hybridization targets were prepared by means of the Affymetrix GeneChip® WT PLUS Reagent Kit using 500 ng of total RNA. The Affymetrix Expression Console Software was used to perform the normalization of the cel files by the Signal Space Transformation (SST) algorithm. Comparison of the treatment groups with vehicle were analyzed in the Affymetrix Transcriptome Analysis Console (TAC) software which calculated the differential gene expression, relative fold change and *p*-values, using the default settings. Of the resulting gene signatures, those meeting a threshold of > 1.5-fold change and a *p*-value <0.05, were uploaded into Ingenuity Pathway Analysis (IPA) for further analysis. qPCR was conducted using the pre-configured TaqMan® Low Density Mouse Immune Array (Life Technologies). cDNA was generated from total RNA using SuperScript VILO Master Mix (Life Technologies). Relative quantification was calculated using the Comparative CT method. For both GeneChip and qPCR data visualization, Clustvis^28^ was used.

### Immunohistochemistry

IHC analysis was performed on tumors from CT26-tumor bearing BALB/c mice (n = 3/group) that had received vehicle or 300 mg/kg p.o. daily of E7046 for 14 d. Tumors were excised, formalin fixed, and embedded in paraffin (FFPE). Sections of FFPE tissue blocks were stained with hematoxylin and eosin, and IHC was performed post antigen retrieval using early-T cell specific rabbit anti-mouse CD3 monoclonal antibody (ThermoFisher Scientific, clone SP7). Percentages of CD3 positive T cells were measured on an Aperio image analyzer using Scan Scope software.

### Statistical analysis

Differences between mice treated with E7046 and control mice were analyzed by the 2-tailed Student *t* test and expressed as the mean ± SEM. For *in vivo* studies involving multiple treatment groups, significance was determined by 2-way ANOVA followed by Tukey's multiple comparisons test. Differences in population frequencies as determined by flow cytometry were analyzed using GraphPad Prism 6 software and significance determined by one-way ANOVA followed by Tukey's multiple comparisons test. *P* < 0.05 was considered significant for all analyses.

## Results

### E7046 reversed the immunosuppressive effects of PGE_2_ on activation and differentiation of human myeloid cells through selective EP4 antagonism

**Figure 1.**
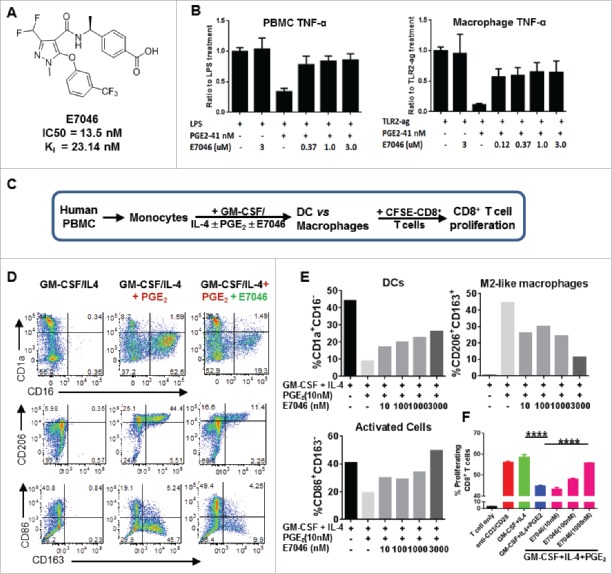
E7046 antagonizes the immunosuppressive effects of PGE_2_ on human monocyte differentiation and activation. A. E7046 structure, IC50 and K_i_ values. B. TNF-α levels measured in LPS-stimulated PBMCs (left graph) or TLR2 agonist-stimulated macrophage co-culture system (right graph) in the presence or absence of PGE_2_ and E7046. Data is presented as mean ± SEM of triplicate values. C. Schematic representation of the human monocyte differentiation assay. D. Representative flow cytometry dot plots showing normal monocyte differentiation to DCs (left panel). PGE_2_ skewed monocyte differentiation from a DC (CD1a^+^CD16^−^) phenotype into a CD1a^−^CD16^+^, CD206^hi^CD163^+^, or CD86^−^CD163^+^, M2-like macrophage phenotype (middle panel). E7046 rescued the DC differentiation of human monocytes (right panel). E. Frequencies of M2-like macrophages, DCs, and activated antigen presenting cells in C plotted by treatment. F. Frequencies of proliferating autologous T cells were determined by gradual loss of CFSE using flow cytometry and plotted by treatment. Representative data from duplicate experiment using cells form one healthy donor shown as mean ± SEM, ****P < 0.0001, One-way ANOVA.

E7046 is a newly developed and highly specific EP4 antagonist with a > 1500 fold selectivity for EP4 relative to EP4 closest homolog, the PGE_2_ receptor EP2 (Fig. 1A, Supplementary Fig. S1).^26^ To evaluate a specific role of PGE_2_-EP4 signaling in pro-tumor myeloid cell activation, we assayed effects of PGE_2_ with or without E7046 and antagonists specific for EP1–3 in a modified BioMap assay system.^29^ This system consists of co-cultures of primary human PBMCs, or purified macrophages, with endothelial cells in conditions that stimulate TCR, or activate either M2TAM or M1 macrophages, allowing a systematic examination of numerous immune readouts including leukocyte proliferation, cytotoxicity, and production of cytokines and chemokines, adhesion molecules, and enzymes (Supplementary Fig. S2A). In these cellular systems neither E7046 nor the EP1–3 antagonists alone altered immune cell functions or survival in the absence of exogenous PGE_2_ (Supplementary Fig. S2B and data not shown). However, a TME-comparable concentration of PGE_2_ significantly suppressed the activation of type 1 macrophages, evidenced by markedly reduced soluble TNF-α and CD40, and promoted type 2 macrophage activity by increasing IL-6 secretion (Supplementary Fig. S2C). Importantly, EP4 antagonist E7046 was a potent inhibitor of PGE_2_ activity in these systems; the EP2 antagonist had much weaker immunomodulatory activity (Supplementary Fig. S2C), and EP1 and EP3 antagonists showed no significant activity (data not shown). TNF-α cytokine secretion was reduced the most by PGE_2_;_’_ E7046 largely restored TNF-α levels in both PBMC and macrophage systems at the concentrations tested (Fig. 1B). Compared to macrophage systems, much less pronounced changes were detected for PGE_2_ and E7046 in TCR-stimulated co-culture systems (second panel of Supplementary Figure S2B,C). The results together indicate a specific role of PGE_2_-EP4 signaling among the 4 EP receptors in promoting the activation of pro-tumor M2-like macrophages and concomitant suppression of anti-tumor M1-like macrophages.

Since PGE_2_ levels correlate with accumulation of immunosuppressive MDSCs and M2TAMs in human tumors and^4^ we next examined the effects of PGE_2_-EP4 signaling in the differentiation and function of monocytes toward antigen-presenting cells, using human monocytes in the presence GM-CSF and IL-4 (Fig. 1C). PGE_2_ (10 nM) showed potent inhibitory activity on GM-CSF/IL-4-induced DC formation. E7046 reduced in a dose-dependent manner the PGE_2_-biased monocyte differentiation toward CD1a^−^CD16^+^ macrophages and promoted differentiation toward CD1a^+^CD16^−^ DCs (Fig. 1D, E). PGE_2_ inhibited expression of the activation marker CD86 and promoted expression of CD163 and CD206 markers, both of which are characteristically associated with the M2-like phenotype (CD163^+^CD206^+^CD86^−^); this effect was dose-dependently inhibited by E7046 (Fig. 1D, E). Furthermore, PGE_2_-differentiated human monocytes exerted an immunosuppressive function toward autologous T cell proliferation in response to TCR stimulation, and this effect was also reduced by E7046 in a dose-dependent manner (Fig. 1F). Together, these results demonstrate that selective pharmacological blockade of EP4 signaling by E7046 was able to largely abolish PGE_2_-mediated pro-tumor myeloid cell differentiation and immunosuppressive activities.

### Blockade of EP4 signaling promotes anti-tumor DC differentiation and slows tumor growth in mice

**Figure 2.**
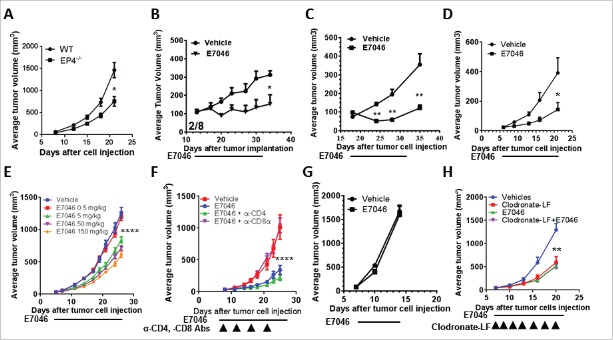
*In vivo* genetic or pharmacological blockade of EP4 results in anti-tumor activity in both a (T)cell and myeloid cell-dependent manner. A. Delayed tumor growth in EP4^−/−^ mice. Growth of s.c. inoculated LLC lung cancer in mice that expressed wild type (EP_4_^flox/flox^), or homozygous (Cre-ER x EP_4_^flox/flox^) deletion in EP4. *P = 0.003, Student's t test; n = 9–10. B. Representative results of EP4 antagonism by E7046 effect on subcutaneous Sal/N fibrosarcoma tumors (n = 8). By the end of treatment, 2 of 8 mice were tumor-free. C. Representative graph of E7046 activity on Pan02 mouse pancreatic cancer s.c. model (n = 5). D. Representative graph of E7046 activity on EMT6 mouse breast cancer orthotopic model (n = 7). E. Dose-dependent E7046 activity against breast cancer 4T1 s.c. model (n = 10). F. CD8^+^ T cell-dependent anti-tumor activity of E7046. CD8^+^ T or CD4^+^ T cells were depleted in CT26 tumor-bearing mice by i.p. administration of anti-CD8 or anti-CD4 antibodies (n = 11). G. Lack of anti-tumor activity of E7046 in CT26 tumor-bearing nude mice. H. Effect of myeloid cells in the anti-tumor activity of E7046. Comparable anti-tumor activities were detected in macrophage-depleted CT26 tumor-bearing mice (treated with clodronate-containing liposomes) as with mice treated with E7046. E7046 was orally administered daily at a dose of 150 mg/kg, while antibodies and liposomes were administered i.p. as shown in the graphs. * *P* < 0.05; **, *P* < 0.01; ***, *P* < 0.001; ****, *P* < 0.0001; 2- tailed student's t-test.

To assess whether EP4 antagonism by E7046 has similar specific activity on mouse myeloid cells, we used murine BM-derived haematopoietic cells induced to differentiate into CD11c-expressing DCs (Supplementary Fig. S3A). E7046 antagonism of PGE_2_ effects on BM cell differentiation rescued T cell proliferation in response to TCR activation in a dose-dependent manner (Supplementary Fig. S3B). Similar to the observations in human co-culture systems, when we compared the effects of EP1-EP3 antagonists with E7046 on mouse BM cell differentiation, only EP4 antagonism rescued the CD11c^+^ DC differentiation from BM cells that PGE_2_ exposure suppressed. This indicated that PGE_2_ mediates its inhibitory effects on mouse myeloid cell differentiation toward DC through EP4 (Supplementary Fig. S3C,D). Furthermore, the myeloid-modifying effects of PGE_2_ could be replicated by the addition of culture supernatants from PGE_2_-producing CT26 and 4T1 mouse cell lines (Supplementary Fig. S3E, F). Addition of E7046 to these supernatants also sufficed to rescue DC differentiation from BM cells (Supplementary Fig. S3E), suggesting that PGE_2_ was the major soluble inhibitory factor secreted by the cancer cells. Finally, we implanted PGE_2_-producing Lewis lung carcinoma (LLC) tumors into EP4 inducible conditional knockout mice in which removal of EP4 was achieved by crossing the EP4^flox/flox^ mice with ER-cre transgenic mice followed by tamoxifen administration. Fig. 2A shows a significant delay in the growth of LLC tumors implanted in EP4 receptor-deficient compared with wild type control mice, indicating that PGE_2_-EP4 signaling in host stromal cells plays an important role in promoting tumor progression.

Taken together, these data demonstrate that PGE_2_ signaling through EP4 plays a dominant immunosuppressive role in myeloid cell differentiation and activation in both human and mouse cells and that E7046, by selectively blocking EP4, inhibits these PGE_2_ activities.

### E7046 displays significant anti-tumor activity in a myeloid- and CD8^+^ T cell-dependent manner

Given the important role of myeloid cells in defining a pro- or anti-tumor immune microenvironment, we next assessed the anti-tumor activity of E7046 in syngeneic mouse cancer models. E7046 has a circulating half-life of approximately 4 hours in mice; daily oral administration of 150 mg/kg E7046 was found to inhibit the growth of multiple syngeneic tumor models: fibrosarcoma SaI/N, pancreatic PAN02, colon CT26, and breast EMT6 and 4T1 (Fig. 2B-E). The highest anti-tumor activity, accompanied by 25% tumor complete regression, was observed in the SaI/N model (Fig. 2B). This anti-tumor activity was dose-dependent (Fig. 2E) and relied on T cells, since tumor-bearing nude mice did not respond to E7046 treatment (Fig. 2G). To confirm and refine this result, CD8^+^ T cell depletion, but not CD4^+^ T cell depletion, completely abrogated the anti-tumor effect of E7046 in immunocompetent mice (Fig. 2F). Furthermore, macrophage depletion by clodronate-containing liposomes impaired tumor growth, but addition of E7046 had no further effect, suggesting that myeloid cells, especially macrophages and MDSC, were the primary cellular targets of E7046 *in vivo* (Fig. 2H). Consistent with the immune-mediated anti-tumor activity, E7046 at concentrations up to 100 µM, did not show any direct anti-proliferative activity against cancer cells of human or mouse origin, or of human endothelial cells *in vitro* (Supplementary Fig. S4). These results indicate a broad anti-tumor efficacy of EP4 blockade by E7046, which is mediated by both myeloid and CD8^+^ T cells.

### E7046 modulates myeloid cells in tumors and thereby shapes the adaptive immune response

**Figure 3.**
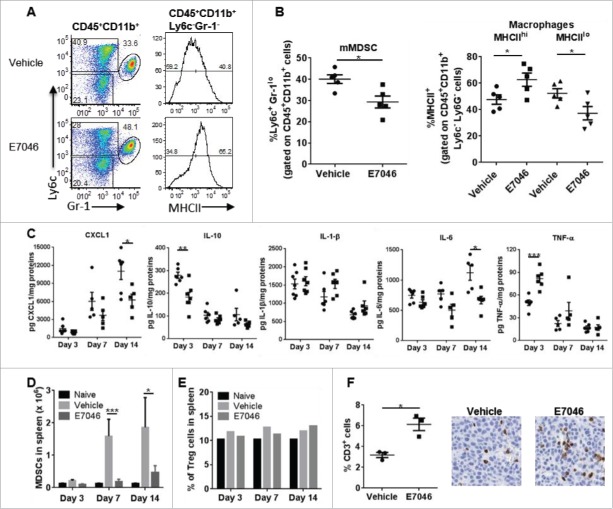
E7046 treatment reduces the intratumoral immunosuppression and promotes anti-tumor immune responses. A. E7046 treatment for 7 d alters myeloid cell phenotype. Single cell suspensions of digested CT26 tumors isolated from E7046 or vehicle-treated mice were analyzed by flow cytometry. Representative dot plots (left panel) of Ly6c and Gr-1-expressing CD11b^+^ cells gated on CD45^+^ cells and histograms (right panel) of MHCII-expressing Ly6c^−^Gr-1^−^ CD11b^+^CD45^+^ cells. B. Frequencies of Ly6c^+^Gr-1^lo^ mMDSC (left graph) and MHCII high or MHCII low-expressing macrophages in A. Data are presented as mean ± SEM of n = 5; * *P* < 0.05. C. Cytokines were measured by MSD reader in CT26 tumor lysates from mice treated with vehicle (filled circles) or E7046 (filled squares) for 3, 7, or 14 d. Data are expressed as mean ± SEM of n = 5; * *P* < 0.05; **, *P* < 0.01; ***, *P* < 0.001. D, E. Splenocytes isolated from naïve or CT26 tumor-bearing mice treated for 3, 7, or 14 d with E7046 or vehicle were counted by hemacytometer and analyzed by flow cytometry. Representative graph showing the number of splenic MDSCs (CD11b^+^Gr-1^+^) (D) and frequencies of Tregs (CD4^+^CD25^+^) (E). Data are expressed as mean ± SEM of n = 5–7, * *P* < 0.05; ***, *P* < 0.001. F. CT26 tumors isolated from mice treated for 14 d with E7046 or vehicle were examined by IHC for the presence of T cells. Frequencies of CD3^+^ T cells in E7046 compared with vehicle-treated mice are shown on the left graph and representative IHC images of tumor sections stained for CD3 marker are shown on the right. Data are presented as mean ± SEM of n = 3, * *P* < 0.05.

The dual myeloid- and CD8^+^ T cell–dependent anti-tumor activity of E7046 is consistent with the finding that E7046 promoted DC development and function and inhibited M2 macrophage development and function *in vitro* (Fig. 1 and Supplementary Fig. S2). To understand the *in vivo* immunomodulatory activity of E7046, we characterized the immune cell composition of tumors from CT26 tumor-bearing mice treated with E7046 or vehicle. We found that, compared with vehicle, E7046 treatment of 7 d significantly reduced the proportions of intra-tumoral CD45^+^CD11b^+^Ly6c^+^Gr-1^lo^mMDSC and increased those of MHCII^hi^ M1-like macrophages while also decreasing MHCII^lo^ M2-like macrophages (Fig. 3A,B). Analysis of TME cytokines revealed dynamic changes in levels of pro- and anti-tumor cytokines and chemokines. Compared to vehicle treatment, E7046 increased the myeloid cell-derived anti-tumor cytokine TNF-α, and reduced the levels of pro-tumor CXCL1, IL-10, and IL-6 (Fig. 3C). While TNF-α levels increased and IL-10 levels decreased early after E7046 administration, on day 3, IL-6 and CXCL1 levels were reduced on day 14. No significant changes in levels of IL-1β were noted. In the spleen, administration of E7046 for 14 d significantly decreased MDSC numbers (Fig. 3D), but did not alter percentages of Treg cells (Fig. 3E). In the tumors, numbers of T cells were significantly increased after 14 d of E7046 treatment (Fig. 3F). These data indicate that *in vivo* the effects of E7046 in EP4 signaling blockade are both systemic, affecting the myeloid cells in both tumor and spleen, and dynamic: reducing the intra-tumoral immunosuppressive MDSCs and M2-like TAMs relatively early after treatment initiation, and promoting intra-tumoral T cell accumulation later during treatment. In addition, E7046 influences the function of TME cells by favoring production of anti-tumor cytokines. These results further implicate immune modulation in the anti-tumor mechanism of E7046 and confirm T cell involvement in the anti-tumor response.

### Simultaneous targeting of PGE_2_-EP4 and Treg cells by E7046 + E7777 combination restores anti-tumor immunity

**Figure 4.**
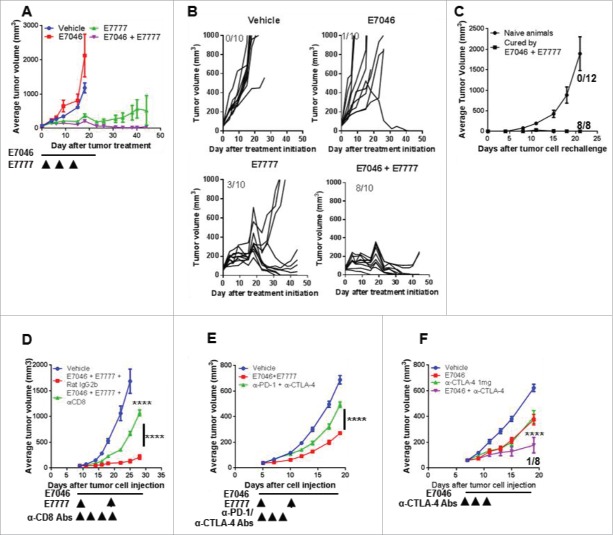
Combination of E7046 + E7777 has potent and immune-dependent anti-tumor activity. A. Anti-tumor activity of E7046 + E7777 on s.c. H22 tumors. E7046 was administered p.o. as 100 mg/kg and E7777 i.v. as 125 µg/kg on the indicated schedules. B. Individual tumor growth graphs for H22 tumor-bearing mice (n = 10). Number of tumor-free per total number of mice is shown on the top left corner of each panel. C. H22 tumor growth plot of re-challenged tumor-free animals from E7046 + E7777 combination treatment and sex and age-matched naïve mice (n = 8–12). D. Anti-tumor activity of E7046 + E7777 is largely dependent on CD8^+^T cells. CT26 tumor-bearing mice were treated with E7047at 150 mg/kg + E7777 at 125 µg/kg, or E7046 + E7777 + anti-mouse CD8 antibodies at 0.250 mg/kg, or vehicle and isotype controls for the indicated duration (n = 5). E. Anti-tumor activity of E7046 + E7777 compared with anti-PD-1 + anti-CTLA-4 antibodies in s.c. 4T1 tumors (n = 10). E7046 was dosed p.o. at 150 mg/kg, daily for 14 d. E7777 was administered as 2 i.v. injections at 2.5 µg/mouse, once a week. Anti-PD-1 and anti-CTLA-4 antibodies and isotype controls (1 mg/mL) were administered i.p. as indicated for a total of 300 µg each. F. Efficacy of E7046 + anti-CTLA-4 antibodies in s.c. 4T1 tumors. Compounds were administered as in E. Data are presented as mean ± SEM of n = 8, with 1/8 rendered tumor-free by the combination. ****, *P* < 0.0001; 2-way ANOVA.

Because E7046 did not impact Treg cell populations (Fig. 3E), we thought to combine it with the Treg-reducing agent E7777 to modulate myeloid cells and Tregs simultaneously. Similarly to the Treg-reducing effect of IL-2-diphtheria toxin fusion protein in patients,^22,23^ intravenous administration of E7777 resulted in a significant reduction in numbers of Tregs, but not CD8^+^ T cells, in mouse spleen (Supplementary Fig. S5). To interrogate the effect of the E7046 and E7777 combination, we chose the hepatocellular carcinoma H22 model, which is rich in both macrophages and Treg cells. Administration of E7777 alone on a Q7Dx3 schedule was effective and rendered 30% of the mice tumor-free, while 100 mg/kg of E7046 alone showed limited activity; only 10% of the treated mice had experienced a complete regression at the end of the study (Fig. 4A,B). Importantly, treatment of H22 tumor-bearing mice with E7046 + E7777 using the same drug doses and schedules as each single agent, was highly effective and rendered 80% of the mice tumor-free (Fig. 4A,B). These tumor-free mice were kept for an additional 30 d without further treatment. No tumor growth was detected in any mice, indicating a durable tumor regression resulting from the combination treatment. Subsequent re-challenge of the combination-cured mice with live H22 cells in the absence of any further treatment resulted in complete tumor rejection, indicating that E7046 + E7777 combination treatment induced effective immunological memory in those animals. In contrast, in all naïve control mice implanted with H22 tumor cells at this time, tumors grew aggressively (Fig. 4C). The combination of E7046 and E7777 was further tested in the moderately immunogenic CT26 and poorly immunogenic 4T1 models. In the CT26 model, combination therapy using the same drug administration regimen resulted in a nearly complete tumor growth inhibition. This activity was largely abolished by CD8^+^ T cell depletion using anti-CD8 antibodies (Fig. 4D). In the 4T1 model, where anti-CTLA-4 antibodies alone or a combination of anti-CTLA-4 and anti-PD-1 antibodies had shown only very limited activity, the combination of E7046 and E7777 was clearly more active than the checkpoint blockade combination (Fig. 4E). Treg cell reduction has been identified as a major anti-tumor activity of anti-CTLA-4 antibodies in preclinical models.^24,25^ Indeed, we observed a combined anti-tumor effect of E7046 and anti-CTLA-4 antibodies compared with each agent alone in this same 4T1 model accompanied by 12.5% tumor-free mice (Fig. 4F), providing additional evidence that EP4 antagonism combined with a Treg cell reduction treatment is a functional strategy to enhance anti-tumor activity. Collectively, these results demonstrate for the first time that combined EP4 blockade and Treg reduction is an effective means of controlling tumor growth and even of rejecting established tumors.

### EP4 signaling blockade by E7046 and Treg cell reduction by E7777 synergistically alter both TME and peripheral anti-tumor immunity

**Figure 5.**
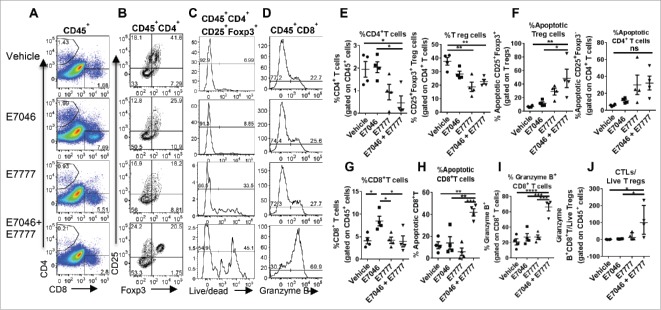
Combination of E7046 + E7777 markedly induces apoptosis of intratumoral Tregs and activation of CD8^+^ (T)cells in the TME. CT26 tumors were isolated 24 hours after the second dosing of E7777, which was also after 7 d of E7046 dosing. Tumors were processed for flow cytometry analysis of T cell populations. A, B. Representative dot plots of intratumoral frequencies of CD4^+^, CD8^+^ T lymphocytes (A), and CD25^+^Foxp3^+^ Treg cells (B). C. Histograms of apoptotic intratumoral CD25^+^Foxp3^+^ Treg cells. D. Histograms of intratumoral granzyme B^+^ CD8^+^ T lymphocytes. E. Quantification of intratumoral CD4^+^ T lymphocytes and Treg cells. F. Analysis of the frequencies of apoptotic Treg cells compared with CD25^+^Foxp3^−^ CD4 T cells in all tumors. G. Quantification of frequencies of intratumoral CD8^+^ T lymphocytes. H. Analysis of the frequencies of apoptotic CD8^+^ T lymphocytes. I. Graph showing frequencies of granzyme B^+^ CD8^+^ T cells. J. Ratio of granzyme B^+^ CD8^+^ T cells to live Treg cells. Data are presented as mean ± SEM of n = 4; * *P* < 0.05; **, *P* < 0.01; ***, P < 0.001; One-way ANOVA.

To understand the mechanism of the anti-tumor immune activity of the E7046 + E7777 combination, we performed cellular immunotyping of CT26 tumors and spleens isolated from treated mice by flow cytometry (Fig. 5, Supplementary Fig. S5). Both types of tissues were isolated 24 hours after the animals had received the second dose of E7777, which was after 7 d of dosing with E7046.

As expected, the CD4^+^ T cell frequencies were decreased in the TME of animals treated with E7777 or the combination of E7046 + E7777, as a result of reduced frequencies of intra-tumoral Treg cells (Fig. 5A,E). The reduction of Tregs was associated with significantly increased apoptosis of these cells, an effect less evident in non-Treg CD25^+^Foxp3^−^CD4^+^ T cells (Fig. 5B,C,F). In line with the result in Fig. 3F, CD8^+^T cell percentages were significantly higher in response to E7046 alone (Fig. 5G). Consistent with the enhanced pharmacological efficacy of the combination, we detected significantly higher frequencies of granzyme B^+^CD8^+^ T cells in the tumors of combination-treated mice compared with vehicle or single agent-treated groups (Fig. 5D,I). Given the CD8^+^ T cell dependency of the anti-tumor effect of the combination (Fig. 4D), we expected enhanced percentages of intra-tumoral CD8^+^ T cells in response to combination treatment. Further analysis found that a large proportion of CD8^+^ T cells in the combination treatment group was positive for the live/dead dye while CD8^+^ T cells in the E7777 single agent-treated group were minimally positive for the live/dead dye. This indicated that the CD8^+^ T cells in the combination group were bona fide functioning CTLs dying while exerting their killing activity (Fig. 5H,I). The ratio of intra-tumoral granzyme B^+^CD8^+^ T cells (CTLs)/live Treg cells was also significantly higher in the group receiving combination treatment than in the vehicle control and single-agent groups, strongly indicative of an ongoing anti-tumor immune response (Fig. 5J).

**Figure 6.**
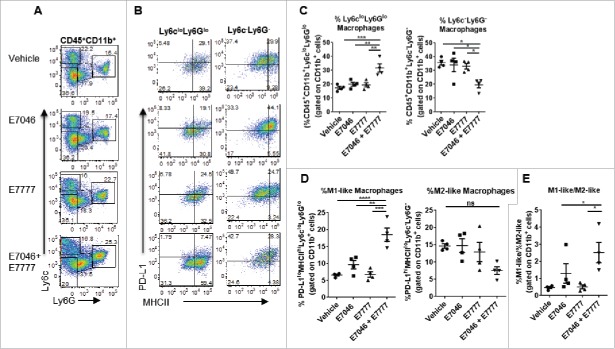
E7046 + E7777 combination alters myeloid cell immunosuppression in the TME. CT26 tumors were isolated 24 hours after the second dose of E7046, which was after 7 d of E7046 dosing. Tumors were processed for flow cytometry analysis of myeloid cell populations. A. Representative dot plots depicting Ly6c and Ly6G expression of intratumoral CD11b^+^ CD45^+^ cells. B. Representative dot plots showing intra-tumoral frequencies of PD-L1^lo^MHCII^hi^ (M1-like) gated on Ly6c^lo^Ly6G^lo^ macrophages (left panel), and PD-L1^hi^MHCII^lo^ (M2-like) gated on Ly6c^−^Ly6G^−^ macrophages (right panel). C. Quantification of frequencies of intratumoral Ly6c^lo^Ly6G^lo^ macrophages (left) and Ly6c^−^Ly6G^−^ macrophages (right), gated on CD11b^+^ cells. D. Quantification of the frequencies of M1-like, PD-L1^lo^MHCII^hi^ TAMs (left), and M2-like, PD-L1^hi^MHCII^lo^ TAMs (right) gated on CD11b^+^ cells. E. Ratio of M1-like to M2-like macrophages. Data are represented as mean ± SEM of n = 4, * *P* < 0.05; **, *P* < 0.01; One-way ANOVA.

Analysis of the intra-tumoral CD11b^+^ myeloid cells showed that the M1-like Ly6c^lo^-expressing macrophages acquired low expression of the granulocytic marker Ly6G in response to E7046 + E7777 combination, while Ly6c^hi^Ly6G^−^ mMDSC frequencies slightly decreased and Ly6c^lo^Ly6G^hi^ gMDSC frequencies increased to some extent (Fig. 6A and data not shown). Thus, based on Ly6c and Ly6G expression, we could distinguish 2 macrophage populations, the M1-like Ly6c^lo^Ly6G^lo^ which was significantly better represented in the combination treatment group, and M2-like Ly6c^−^Ly6G^−^ TAMs which were reduced by E7046 + E7777 combination compared with vehicle treatment (Fig. 6B,C). These cells were analyzed separately for MHCII and PD-L1 expression. The percentage of PD-L1^lo^ and MHCII^hi^ M1-like TAMs was significantly higher in the combination treatment compared with vehicle group while 30–50% of M2-like TAMs were PD-L1^hi^MHCII^lo^ regardless of treatment (Fig. 6D). The ratio of M1-like/M2-like macrophages in the tumor was significantly increased by E7046 + E7777 treatment compared with vehicle or single agent treatment (Fig. 6E) indicating that the E7046 + E7777 combination is able to alter the TAM populations by favoring the anti-tumor M1-like over pro-tumor M2-like TAMs.

### EP4 signaling blockade by E7046 and Treg reduction by E7777 act synergistically to generate a gene expression signature consistent with anti-tumor immune activation

**Figure 7.**
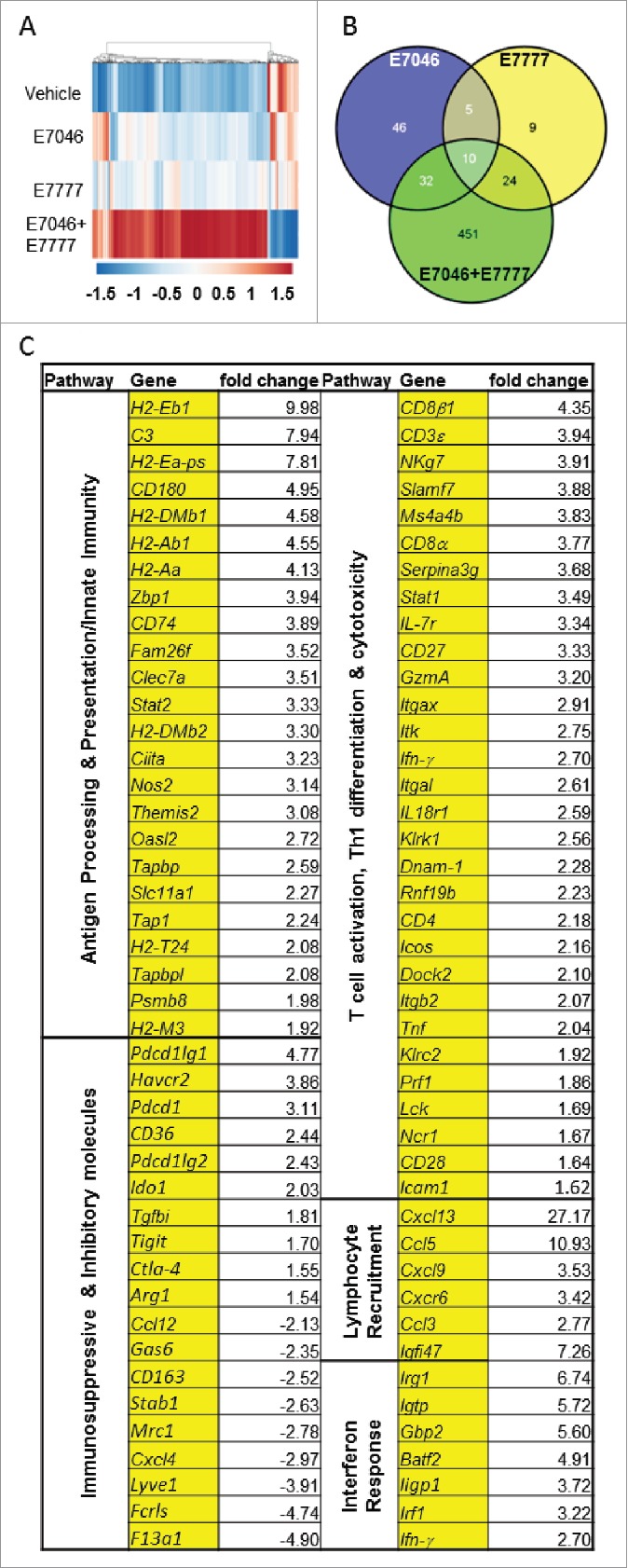
E7046 + E7777 combination induces an immune gene profile consistent with acute inflammation and tumor rejection. E7046 (150 mg/kg) was administered p.o. daily for 20 days, and E7777 was injected i.v. as 3 doses of 125 µg/kg every 7 d. CT26 tumors were isolated 24 hours after the third dosing of E7777 which was after 20 d of E7046 dosing, then processed for gene expression analysis. A. Cluster analysis of microarray gene expression representing genes significantly upregulated (red) or downregulated (blue) in TME by treatment (≥ 1.5-fold change, P ≤ 0.05). B. Venn diagram depicting the number of genes expressed as a result of E7046, E7777, or the combination, and commonly regulated genes. C. Fold change in mRNA levels of selected gene sets from E7046 + E7777 compared with vehicle groups are shown with associated biologic activities.

To further investigate the mechanism, expression of immune genes was determined by microarray and TaqMan® Low Density Array (TLDA) in CT26 tumors isolated from control mice and mice that had received daily oral doses of E7046 for 20 days, or 3 doses of E7777 once every 7 days, or a combination of both. We considered as significant gene expression changes ≥ 1.5-fold and *P*<0.05. RNA expression results show that, while E7046 or E7777 alone elicited modest gene expression changes compared with vehicle control, E7046 + E7777 treatment strongly upregulated numerous genes involved in antigen presentation, adhesion, Th1 cell differentiation, activation of cytotoxic mechanisms, Cxcr3 and Ccr5 ligand chemokines, but also tumor and myeloid-produced factors and known T cell co-inhibitory receptors (Fig. 7, Supplementary Fig. S6). The modest gene expression changes in response to EP4 antagonism or Treg cell reduction alone was expected given that neither E7046 nor E7777 are transcriptional regulators; however, their combination showed synergistic activity on gene expression, both in the number of genes and the amplitude of the response (Fig. 7A,B). Pathway analysis of the altered immune gene expression revealed that the genes significantly up- or downregulated in response to treatment belong to multiple pathways involving immune functions (Supplementary Fig. S6A). Gene expression analysis indicated M1-like macrophage activation: upregulation of genes encoding MHC type I and II proteins (*H2-T24, H2-Eb1, H2-M3*), antigen processing and presentation machinery: class II trans-activator (*Ciita*), transporter 1 ATP-binding cassette (*Tap1*), proteasome subunit β type 8 *(Psmb8*), TAP binding protein (*Tapbp*), solute carrier family 11a1 (*Slc11a1*), innate immune sensing proteins such as Z-DNA binding protein 1 (*Zbp1*), and functional molecules such as inducible nitric oxide synthase 2 (*Nos2*) (Fig. 7C, Supplementary Fig. S6B). These effects were paralleled by a concerted decrease in M2TAMs and the associated immunosuppressive functions marked by downregulation of scavenger receptor gene *CD163*, Fc receptor-like S (*Fcrls*), stabilin 1 (S*tab1*), mannose receptor (M*rc1*), and other M2TAM markers and secreted proteins, including lymphatic vessel endothelial hyaluronan receptor 1 (*Lyve1*), and coagulation factor XIII A subunit (*F13a1*)^30-33^ (Fig. 7C). Of note, *Ptgs2*, which encodes Cox2 (and thus, controls the cascade leading to PGE_2_ production) was not regulated by E7046 or by the combination (Supplementary Fig. S6B). The E7046 + E7777 combination also enhanced transcription of markers of M1-like macrophage and DC activation (*CD40*), numerous NK activating receptors such as natural killer cell group 7 sequence (*NKg7*), killer cell lectin-like receptor subfamily k 1 (*Klrk1*) encoding NKG2D, CRACC (*Slamf7*), and STAT1 as well as IFN-γ target genes involved in DC maturation and NK cell activation. In the same class, C-type lectin domain family 7a (C*lec7a*), family with sequence similarity 26 (*Fam26f*), oligoadenylate synthetase 2 (O*asl2*), and NK-lytic associated molecule NKLAM (*Rnf19b*) (Fig. 7C, Supplementary Fig. S6B).^34-36^ Finally, E7046 + E7777 treatment induced immune cell activation and effector function within the tumor, as evidenced by increased mRNA expression of T cell receptors (*CD8α, CD8β*), inflammatory chemokines (*Ccl5, Ccl3, Cxcl9, Cxcl10, Cxcl11, Cxcl13*), CTL promoters such as membrane-spanning 4-domains, subfamily A, member 4B (*Ms4a4b*), Th1 cytokines and transcription factors (*Ifn-γ, IL-2, Tnf, Tbx21*), classical IFN-γ stimulated genes (*Stat1, Irf1, Igtp, Fam26f*), adhesion molecules (*Icam1, Lfa-1*), T cell activation markers (*Icos, CD40L, CD28, CD27*), and molecules directly involved in tumor cell recognition and cytotoxic effector function (*Granzyme A, Perforin, Dnam-1, Nklam*) (Fig. 7C, Supplementary Fig. S6B).^37,38^

Overall, transcriptional analysis suggested a potent switch from chronic to acute inflammation mediated by activation of the Stat-1/Irf-1 pathway, similar to an immune response to intracellular viral pathogens. Notably, the powerful intratumoral immune activation that we observed in response to the combination treatment also caused upregulation of genes encoding immunosuppressive molecules such as P*dcd1* (encoding PD-1), *Havcr2* (Tim-3), *Ido1, Pdcd1lg1* (PD-L1), *Pdcd1lg2* (PD-L2), and *Ctla-4*. MDSC markers *CD36* and A*rg1* were upregulated to a lesser extent (Fig. 7C, Supplementary Fig. S6B). Together with the above described TME immune cellular profiling results, the molecular profiling data indicated a highly synergistic and effective immunomodulatory activity in the TME by the E7046 and E7777 combination, which offers a mechanistic explanation for the potent anti-tumor activity and long lasting immune memory response.

### Discussion

We have shown that PGE_2_-EP4 signaling plays a specific and important role in driving pro-tumor myeloid cell differentiation and function, and that pharmacological blockade of EP4 signaling by E7046, a novel EP4 antagonist in early clinical development, is able to largely reverse PGE_2_-induced myeloid immunosuppression and thus promote anti-tumor immune responses. The specific role of EP4, but not other EP receptors, was unexpected because both EP2 and EP4 have been reported by others to contribute to PGE_2_-mediated activity on immune cells.^39^ This discrepancy may be explained by differential cellular and tissue expression of EP2 and EP4, and also by the incomplete specificity of agents used in earlier studies. Our results are consistent with a recent finding that selective deletion of EP4 in macrophages effectively inhibited intestinal adenoma development and growth in colon cancer-prone APC^min/+^ mice.^5^ The significant reduction of tumor growth in inducible EP4^−/−^ compared with WT mice supports the important role of host cell EP4 in tumor progression. Importantly, though myeloid cells appear to be the major direct target cell population for E7046, *in vivo* anti-tumor activity of E7046 requires CD8^+^ cytolytic T cells but not CD4^+^ T cells, suggesting a unique mechanism of action of E7046 in which reduced myeloid cell-based immunosuppression facilitates a cytolytic CD8^+^ T cell-dependent anti-tumor activity. Accumulation of T cells in E7046-treated compared with vehicle-treated tumors supports this notion. Further studies will be required to show the full extent of *in vivo* activation of these cells in response to E7046.

Because E7046 affects M2TAMs and mMDSCs but not Treg cells, we hypothesized that by combining EP4 signaling blockade with Treg cell reduction we would obtain an enhanced immunomodulatory anti-tumor effect. Indeed, we identified a strong synergy in inducing tumor regression and/or growth control in multiple animal models when both agents were administered simultaneously. Immunophenotyping at both cellular and molecular levels demonstrated a strong synergistic effect of the combination in the initiation of acute inflammation and immune activation for an effective anti-tumor response. The granzyme B^+^ CTL effectors detected in CT26 tumors which are contributing to this response also stained positive for live/dead dye which is an indication of their short-lived nature as well as their possible increased susceptibility to E7777 targeting. Notably, the combination of E7046 + E7777 was more effective than the combination of anti-PD-1+anti-CTLA-4 dual checkpoint blockade therapy in the “cold” 4T1 breast cancer model, which is characterized by strong myeloid cell-mediated immunosuppression and is typically refractory to T cell checkpoint inhibitors. This finding indicates that PGE_2_-EP4 signaling blockade coupled to Treg reduction would be a synergistic anti-tumor combination in general. In support of this notion, combination of E7046 and anti-CTLA-4 antibodies, known to reduce Treg cells in both cancer patients and preclinical models,^24,25,40^ was superior to either agent alone.

E7046 + E7777 treatment activated the innate immune system which led to an “inflamed” TME evidenced by expression of genes encoding Ccl5/RANTES, macrophage inflammatory protein-1α (MIP-1α/Ccl3), Cxcl9, Cxcl10, and Cxcl11, previously found associated with tumor destruction.^41^ These chemokines are well known for their anti-tumor roles in recruiting and activating a wide array of immune cells mediated through a common receptor Cxcr3,^41^ also upregulated by the combination. *Cxcl13*, encoding a B and T cell chemoattractant, was the most upregulated chemokine gene in response to combination treatment. Although previously associated with a Th2 response,^42^ a recent study has found that expression of *Cxcl13* is strongly associated with B, follicular helper T, Th1, and cytotoxic T cells, and also correlates with a significantly prolonged disease-free survival time in both preclinical models and colorectal cancer patients.^43^ E7046 + E7777 downregulated the expression of *Ccl12*, a chemokine gene reported to be expressed in MHCII^lo^TAMs^30^ and the *pf4* gene (platelet factor 4 or Cxcl4), which hints at platelets as additional targets of the combination. Interestingly, like PGE_2_, CXCL4 has been shown to bias human monocyte differentiation into CD1a^−^ DCs which were less capable than CD1a^+^ DCs in stimulating allogeneic T cells to proliferate.^44^ It is conceivable then that EP4 antagonism combined with Treg reduction is able to block myeloid cell differentiation at multiple levels.

While cellular and molecular immunophenotyping demonstrated a robust reprogramming of both innate and adaptive anti-tumor immunity in response to E7046 + E7777 combination therapy, it also induced expression of genes encoding immunosuppressive molecules such as ido1, arg1, PD-1, PD-L1, and Tim3. Though PD-1 expression has not always been associated with the exhausted T cell phenotype,^45^ our results suggest that exhausted T cells and their ligand-expressing immunosuppressive cells are present within the treated tumors and thus, addition of checkpoint inhibitors to this treatment should further enhance the anti-tumor response. In this context, EP4 was found expressed on terminally exhausted CTLs and its expression has been correlated with PD-1 expression in conditions involving persistent antigen stimulation such as chronic viral infections and cancer.^10,46,47^ The co-expression of EP4 and PD-1 on exhausted T cells raises the intriguing possibility that dual blockade of EP4 and PD-1 may limit T cell exhaustion. PD-L1 expression on TAMs as well as on tumor cells has been found associated with resistance to immunotherapies but is also dependent on CD8^+^ T cell presence.^48^

In conclusion, we have shown here that a specific EP4 blockade by E7046 reversed the PGE_2_-induced myeloid-mediated immunosuppression and that E7046 + E7777 synergistically mitigated both myeloid and Treg-derived immunosuppression within the TME, to promote robust anti-tumor immune responses. Either E7046 alone or E7046 + E7777 combination therapy may benefit cancer patients with tumors enriched in myeloid and Treg cells. These approaches are the subject of on-going further investigation.

## Supplementary Material

KONI_A_1338239_s02.pdf

## References

[1] Wang D, Dubois RN The role of COX-2 in intestinal inflammation and colorectal cancer. Oncogene 2010; 29(6):781-8; PMID:19946329; https://doi.org/10.1038/onc.2009.421 19946329 PMC3181054

[2] Hangai S, Ao T, Kimura Y, Matsuki K, Kawamura T, Negishi H, Nishio J, Kodama T, Taniguchi T, Yanai H PGE2 induced in and released by dying cells functions as an inhibitory DAMP. Proc Natl Acad Sci U S A 2016; 113(14):3844-9; PMID:27001836; https://doi.org/10.1073/pnas.1602023113 27001836 PMC4833254

[3] Hata AN, Breyer RM Pharmacology and signaling of prostaglandin receptors: multiple roles in inflammation and immune modulation. Pharmacol Ther 2004; 103(2):147-66; PMID:15369681; https://doi.org/10.1016/j.pharmthera.2004.06.003 15369681

[4] Kalinski P Regulation of immune responses by prostaglandin E2. J Immunol 2012; 188(1):21-8; PMID:22187483; https://doi.org/10.4049/jimmunol.1101029 22187483 PMC3249979

[5] Chang J, Vacher J, Yao B, Fan X, Zhang B, Harris RC, Zhang MZ Prostaglandin E receptor 4 (EP4) promotes colonic tumorigenesis. Oncotarget 2015; 6(32):33500-11; PMID:26378024 26378024 10.18632/oncotarget.5589 PMC4741781

[6] Snyder DS, Beller DI, Unanue ER Prostaglandins modulate macrophage Ia expression. Nature 1982; 299(5879):163-5; PMID:6287286; https://doi.org/10.1038/299163a0 6287286

[7] Scales WE, Chensue SW, Otterness I, Kunkel SL Regulation of monokine gene expression: prostaglandin E2 suppresses tumor necrosis factor but not interleukin-1 alpha or beta-mRNA and cell-associated bioactivity. J Leukoc Biol 1989; 45(5):416-21; PMID:2651547 2651547

[8] van der Pouw Kraan TC, Boeije LC, Smeenk RJ, Wijdenes J, Aarden LA Prostaglandin-E2 is a potent inhibitor of human interleukin 12 production. J Exp Med 1995; 181(2):775-9; PMID:7836930; https://doi.org/10.1084/jem.181.2.775 7836930 PMC2191857

[9] Ahmadi M, Emery DC, Morgan DJ Prevention of both direct and cross-priming of antitumor CD8+ T-cell responses following overproduction of prostaglandin E2 by tumor cells in vivo. Cancer Res 2008; 68(18):7520-9; PMID:18794140; https://doi.org/10.1158/0008-5472.CAN-08-1060 18794140 PMC2546514

[10] Chen JH, Perry CJ, Tsui YC, Staron MM, Parish IA, Dominguez CX, Rosenberg DW, Kaech SM Prostaglandin E2 and programmed cell death 1 signaling coordinately impair CTL function and survival during chronic viral infection. Nat Med 2015; 21(4):327-34; PMID:25799228; https://doi.org/10.1038/nm.3831 25799228 PMC4505619

[11] Minakuchi R, Wacholtz MC, Davis LS, Lipsky PE Delineation of the mechanism of inhibition of human T cell activation by PGE2. J Immunol 1990; 145(8):2616-25; PMID:1976699 1976699

[12] Katamura K, Shintaku N, Yamauchi Y, Fukui T, Ohshima Y, Mayumi M, Furusho K Prostaglandin E2 at priming of naive CD4+ T cells inhibits acquisition of ability to produce IFN-gamma and IL-2, but not IL-4 and IL-5. J Immunol 1995; 155(10):4604-12; PMID:7594459 7594459

[13] Basingab FS, Ahmadi M, Morgan DJ IFNgamma-dependent interactions between ICAM1 and LFA-1 counteract prostaglandin E2-mediated inhibition of antitumor CTL response. Cancer Immunol Res 2016; 4(5):400-11; PMID:26928462; https://doi.org/10.1158/2326-6066.CIR-15-0146 26928462

[14] Sharma S, Yang SC, Zhu L, Reckamp K, Gardner B, Baratelli F, Huang M, Batra RK, Dubinett SM Tumor cyclooxygenase-2/prostaglandin E2-dependent promotion of FOXP3 expression and CD4+ CD25+ T regulatory cell activities in lung cancer. Cancer Res 2005; 65(12):5211-20; PMID:15958566; https://doi.org/10.1158/0008-5472.CAN-05-0141 15958566

[15] Joyce JA, Pollard JW Microenvironmental regulation of metastasis. Nat Rev Cancer 2009; 9(4):239-52; PMID:19279573; https://doi.org/10.1038/nrc2618 19279573 PMC3251309

[16] Movahedi K, Guilliams M, Van den Bossche J, Van den Bergh R, Gysemans C, Beschin A, De Baetselier P, Van Ginderachter JA Identification of discrete tumor-induced myeloid-derived suppressor cell subpopulations with distinct T cell-suppressive activity. Blood 2008; 111(8):4233-44; PMID:18272812; https://doi.org/10.1182/blood-2007-07-099226 18272812

[17] Mantovani A, Sica A, Allavena P, Garlanda C, Locati M Tumor-associated macrophages and the related myeloid-derived suppressor cells as a paradigm of the diversity of macrophage activation. Hum Immunol 2009; 70(5):325-30; PMID:19236898; https://doi.org/10.1016/j.humimm.2009.02.008 19236898

[18] Allavena P, Sica A, Solinas G, Porta C, Mantovani A The inflammatory micro-environment in tumor progression: the role of tumor-associated macrophages. Crit Rev Oncol Hematol 2008; 66(1):1-9; PMID:17913510; https://doi.org/10.1016/j.critrevonc.2007.07.004 17913510

[19] Luan B, Yoon YS, Le Lay J, Kaestner KH, Hedrick S, Montminy M CREB pathway links PGE2 signaling with macrophage polarization. Proc Natl Acad Sci U S A 2015; 112(51):15642-7; PMID:26644581 26644581 10.1073/pnas.1519644112 PMC4697393

[20] Zou W Regulatory T cells, tumour immunity and immunotherapy. Nat Rev Immunol 2006; 6(4):295-307; PMID:16557261; https://doi.org/10.1038/nri1806 16557261

[21] Salagianni M, Lekka E, Moustaki A, Iliopoulou EG, Baxevanis CN, Papamichail M, Perez SA NK cell adoptive transfer combined with Ontak-mediated regulatory T cell elimination induces effective adaptive antitumor immune responses. J Immunol 2011; 186(6):3327-35; PMID:21317394; https://doi.org/10.4049/jimmunol.1000652 21317394

[22] Wong BY, Ma Y, Fitzwilson R, Dang NH De novo maintenance therapy with denileukin diftitox (Ontak) in a patient with peripheral T-cell lymphoma is associated with prolonged remission. Am J Hematol 2008; 83(7):596-8; PMID:18383317; https://doi.org/10.1002/ajh.21177 18383317

[23] Gritzapis AD, Voutsas IF, Baxevanis CN Ontak reduces the immunosuppressive tumor environment and enhances successful therapeutic vaccination in HER-2/neu-tolerant mice. Cancer Immunol Immunother 2012; 61(3):397-407; PMID:21928125; https://doi.org/10.1007/s00262-011-1113-4 21928125 PMC11029548

[24] Simpson TR, Li F, Montalvo-Ortiz W, Sepulveda MA, Bergerhoff K, Arce F, Roddie C, Henry JY, Yagita H, Wolchok JD, et al. Fc-dependent depletion of tumor-infiltrating regulatory T cells co-defines the efficacy of anti-CTLA-4 therapy against melanoma. J Exp Med 2013; 210(9):1695-710; PMID:23897981; https://doi.org/10.1084/jem.20130579 23897981 PMC3754863

[25] Selby MJ, Engelhardt JJ, Quigley M, Henning KA, Chen T, Srinivasan M, Korman AJ Anti-CTLA-4 antibodies of IgG2a isotype enhance antitumor activity through reduction of intratumoral regulatory T cells. Cancer Immunol Res 2013; 1(1):32-42; PMID:24777248; https://doi.org/10.1158/2326-6066.CIR-13-0013 24777248

[26] Bender AT, Spyvee M, Satoh T, Gershman B, Teceno T, Burgess L, Kumar V, Wu Y, Yang H, Ding Y, et al. Evaluation of a candidate anti-arthritic drug using the mouse collagen antibody induced arthritis model and clinically relevant biomarkers. Am J Transl Res 2013; 5(1):92-102; PMID:23390569 23390569 PMC3560477

[27] Schneider A, Guan Y, Zhang Y, Magnuson MA, Pettepher C, Loftin CD, Langenbach R, Breyer RM, Breyer MD Generation of a conditional allele of the mouse prostaglandin EP4 receptor. Genesis 2004; 40(1):7-14; PMID:15354288; https://doi.org/10.1002/gene.20048 15354288

[28] Metsalu T, Vilo J ClustVis: a web tool for visualizing clustering of multivariate data using Principal Component Analysis and heatmap. Nucleic Acids Res 2015; 43(W1):W566-70; PMID:25969447 25969447 10.1093/nar/gkv468 PMC4489295

[29] Berg L, Hsu YC, Lee JA Consideration of cellualr microenvironment: physiologically relevant co-culture sytems in drug discovery. Adv Drug Deliv Rev 2014; 69-70:190-204; PMID:24524933 24524933 10.1016/j.addr.2014.01.013

[30] Movahedi K, Laoui D, Gysemans C, Baeten M, Stange G, Van den Bossche J, Mack M, Pipeleers D, In't Veld P, De Baetselier P, et al. Different tumor microenvironments contain functionally distinct subsets of macrophages derived from Ly6C(high) monocytes. Cancer Res 2010; 70(14):5728-39; PMID:20570887 20570887 10.1158/0008-5472.CAN-09-4672

[31] Torocsik D, Bardos H, Nagy L, Adany R Identification of factor XIII-A as a marker of alternative macrophage activation. Cell Mol Life Sci 2005; 62(18):2132-9; PMID:16132226 16132226 10.1007/s00018-005-5242-9 PMC11139147

[32] Schledzewski K, Falkowski M, Moldenhauer G, Metharom P, Kzhyshkowska J, Ganss R, Demory A, Falkowska-Hansen B, Kurzen H, Ugurel S, et al. Lymphatic endothelium-specific hyaluronan receptor LYVE-1 is expressed by stabilin-1+, F4/80+, CD11b+ macrophages in malignant tumours and wound healing tissue in vivo and in bone marrow cultures in vitro: implications for the assessment of lymphangiogenesis. J Pathol 2006; 209(1):67-77; PMID:16482496 16482496 10.1002/path.1942

[33] Algars A, Irjala H, Vaittinen S, Huhtinen H, Sundstrom J, Salmi M, Ristamäki R, Jalkanen S Type and location of tumor-infiltrating macrophages and lymphatic vessels predict survival of colorectal cancer patients. Int J Cancer 2011; 131(4):864-73; PMID:21952788 21952788 10.1002/ijc.26457

[34] Kasahara S, Clark EA Dendritic cell-associated lectin 2 (DCAL2) defines a distinct CD8alpha- dendritic cell subset. J Leukoc Biol 2012; 91(3):437-48; PMID:22147811 22147811 10.1189/jlb.0711384 PMC3289397

[35] Malilas W, Koh SS, Srisuttee R, Boonying W, Cho IR, Jeong CS, Johnston RN, Chung YH Cancer upregulated gene 2, a novel oncogene, confers resistance to oncolytic vesicular stomatitis virus through STAT1-OASL2 signaling. Cancer Gene Ther 2013; 20(2):125-32; PMID:23306614 23306614 10.1038/cgt.2012.96

[36] Kozlowski M, Schorey J, Portis T, Grigoriev V, Kornbluth J NK lytic-associated molecule: a novel gene selectively expressed in cells with cytolytic function. J Immunol 1999; 163(4):1775-85; PMID:10438909 10438909

[37] Xu H, Williams MS, Spain LM Patterns of expression, membrane localization, and effects of ectopic expression suggest a function for MS4a4B, a CD20 homolog in Th1 T cells. Blood 2006; 107(6):2400-8; PMID:16293604; https://doi.org/10.1182/blood-2005-08-3340 16293604 PMC1895730

[38] Ramsbottom KM, Hawkins ED, Shimoni R, McGrath M, Chan CJ, Russell SM, Smyth MJ, Oliaro J Cutting edge: DNAX accessory molecule 1-deficient CD8+ T cells display immunological synapse defects that impair antitumor immunity. J Immunol 2014; 192(2):553-7; PMID:24337740; https://doi.org/10.4049/jimmunol.1302197 24337740

[39] Obermajer N, Kalinski P Key role of the positive feedback between PGE(2) and COX2 in the biology of myeloid-derived suppressor cells. Oncoimmunology 2012; 1(5):762-4; PMID:22934275; https://doi.org/10.4161/onci.19681 22934275 PMC3429587

[40] Nancey S, Boschetti G, Cotte E, Ruel K, Almeras T, Chauvenet M, Stroeymeyt K, Moussata D, Kaiserlian D, Flourié B Blockade of cytotoxic T-lymphocyte antigen-4 by ipilimumab is associated with a profound long-lasting depletion of Foxp3+ regulatory T cells: a mechanistic explanation for ipilimumab-induced severe enterocolitis? Inflamm Bowel Dis 2012; 18(8):E1598-600; PMID:22069060; https://doi.org/10.1002/ibd.21927 22069060

[41] Galon J, Angell HK, Bedognetti D, Marincola FM The continuum of cancer immunosurveillance: prognostic, predictive, and mechanistic signatures. Immunity 2013; 39(1):11-26; PMID:23890060; https://doi.org/10.1016/j.immuni.2013.07.008 23890060

[42] Murray PJ, Wynn TA Protective and pathogenic functions of macrophage subsets. Nat Rev Immunol 2011; 11(11):723-37; PMID:21997792; https://doi.org/10.1038/nri3073 21997792 PMC3422549

[43] Bindea G, Mlecnik B, Tosolini M, Kirilovsky A, Waldner M, Obenauf AC, Angell H, Fredriksen T, Lafontaine L, Berger A, et al. Spatiotemporal dynamics of intratumoral immune cells reveal the immune landscape in human cancer. Immunity 2013; 39(4):782-95; PMID:24138885; https://doi.org/10.1016/j.immuni.2013.10.003 24138885

[44] Xia CQ, Kao KJ Effect of CXC chemokine platelet factor 4 on differentiation and function of monocyte-derived dendritic cells. Int Immunol 2003; 15(8):1007-15; PMID:12882838; https://doi.org/10.1093/intimm/dxg100 12882838

[45] Fourcade J, Kudela P, Sun Z, Shen H, Land SR, Lenzner D, Guillaume P, Luescher IF, Sander C, Ferrone S, et al. PD-1 is a regulator of NY-ESO-1-specific CD8+ T cell expansion in melanoma patients. J Immunol 2009; 182(9):5240-9; PMID:19380770; https://doi.org/10.4049/jimmunol.0803245 19380770 PMC3426222

[46] Baitsch L, Baumgaertner P, Devevre E, Raghav SK, Legat A, Barba L, Wieckowski S, Bouzourene H, Deplancke B, Romero P, et al. Exhaustion of tumor-specific CD8(+) T cells in metastases from melanoma patients. J Clin Invest 2011; 121(6):2350-60; PMID:21555851; https://doi.org/10.1172/JCI46102 21555851 PMC3104769

[47] Martinez GJ, Pereira RM, Aijo T, Kim EY, Marangoni F, Pipkin ME, Togher S, Heissmeyer V, Zhang V, Crotty S, et al. The transcription factor NFAT promotes exhaustion of activated CD8(+) T cells. Immunity 2015; 42(2):265-78; PMID:25680272; https://doi.org/10.1016/j.immuni.2015.01.006 25680272 PMC4346317

[48] Spranger S, Spaapen RM, Zha Y, Williams J, Meng Y, Ha TT, Gajewski TF Up-regulation of PD-L1, IDO, and T(regs) in the melanoma tumor microenvironment is driven by CD8(+) T cells. Sci Transl Med 2013; 5(200):200ra116; PMID:23986400; https://doi.org/10.1126/scitranslmed.3006504 23986400 PMC4136707

