# Supplementary material for: EP4 Antagonism by E7046 diminishes Myeloid immunosuppression and synergizes with Treg-reducing IL-2-Diphtheria toxin fusion protein in restoring anti-tumor immunity
Source: Oncoimmunology. 2017 Jun 28;6(8):e1338239. doi: 10.1080/2162402X.2017.1338239 (PMC5593700; doi:10.1080/2162402X.2017.1338239)
Supplement: KONI_A_1338239_s02.pdf [file koni-06-08-1338239-s001.pdf]

SUPLEMENTARY DATA

The supplementary data file contains supplemental materials and methods, and six supplementary figures with figure legends.

SUPPLEMENTARY MATERIAL AND METHODS

Cell growth inhibition assay

Cells were seeded in 96-well tissue culture plates at 500 – 3000 cells/well (seeding density empirically adjusted for cell proliferation rate). Cells were allowed to attach for a minimum of 5 hours prior to addition of serial dilutions of E7046 starting at 100 µM. Three, four, or six days after the treatment, CellTiter-Glo reagent (Promega, Madison, WI) was added to all wells to assess cell proliferation/viability. Luminescence was measured using an Envision microplate reader (Perkin Elmer, Waltham, MA). The IC_{50} values were calculated as the concentration which inhibited cell growth to 50% of the level observed in cells treated with DMSO.

PGE_2 measurements and cell-based EP4 selectivity assay

Murine cell lines 4T1 and CT26 were grown near confluency when medium was replaced with 2%FBS-containing medium for 24 h followed by harvest for PGE_2 measurement. PGE_2 was measured in supernatant using the PGE_2 Assay kit (R&D Systems) and manufacturer's protocol. Data was analyzed using GraphPad Prism software.

The selectivity of E7046 for EP4 or EP2 receptors has been tested using the PathHunter β-arrestin enzyme fragment complementation technology with β-galactosidase (DiscoveRx). In this system, β-galactosidase was split into two inactive complementary fragments, a small donor peptide named ProLink^{TM} expressed fused to the GPCR of interest, and an enzyme acceptor, expressed fused to β-arrestin. Activation of GPCR recruited β-arrestin to the receptor and

brought the two inactive fragments together which restored β-galactosidase activity, detected as chemiluminescent signals using PathHunter detection reagents (DiscoveRx). Custom GPCR profiling for EP4-arrestin-antagonist and EP2-arrestin-antagonist was performed using E7046 and ER-880696 (EP2 antagonist) in the presence of PGE_2 at EC80. First, PGE_2 or vehicle control was tested using the agonist format; PGE_2 was incubated with cells expressing EP4 or EP2 at 37°C for 90 min. Vehicle control measured potential constitutive activity in the absence of ligand. Data was normalized to the maximal and minimal response observed in the presence of PGE_2 and vehicle and the percentage of activity was calculated using the following formula: % Actvity =100% x (mean RLU of test sample —mean RLU of vehicle control) / (mean MAX PGE_2 — mean RLU of vehicle control). RLU = relative luminescence units. Next, an EC80 of 1.2 μM PGE_2 for EP2 and 3.6 nM PGE_2 for EP4 were used to test the antagonist activity of E7046 and ER-880696 on EP4 and EP2 receptors using the antagonist assay format. Data was normalized to the maximal and minimal response observed in the presence of EC80 PGE_2 and vehicle and the percentage inhibition was calculated using the formula: % Inhibiton =100% x (1 — (mean RLU of test sample— mean RLU of vehicle control) / (mean RLU of EC80 PGE_2 — mean RLU of vehicle control)).

Measurement of immunomodulatory effects of PGE_2 and EP1-4 antagonists in cellular co-culture systems

PGE_2 and EP1-4 antagonists were tested for broad immunomodulatory activity in primary human co-culture cellular systems designed to test compound(s) activity on immune cell activation, using a custom assay based on the BioMAP® platform (DiscoveRx). Briefly, the system consists of co-cultures of PBMCs or type 1 or 2 macrophages with endothelial cells in the presence of inflammatory mediators (TLR2 and TLR4 agonists) or TCR and/or BCR stimulation for 24 hours in the presence of PGE_2 (41.1 nM) ± EP1-4 antagonists (3 μM). Levels of protein readouts in the supernatants (cytokines, chemokines, enzymes, adhesion molecules)

were measured and used to generate activity profiles (data sets normalized to baseline profiles in the absence of the test compounds). Activity profiles generated by E1-4 antagonists in the presence of PGE_2 were compared to obtain information about the role of each of the EP receptors in the immunosuppressive activity of PGE_2.

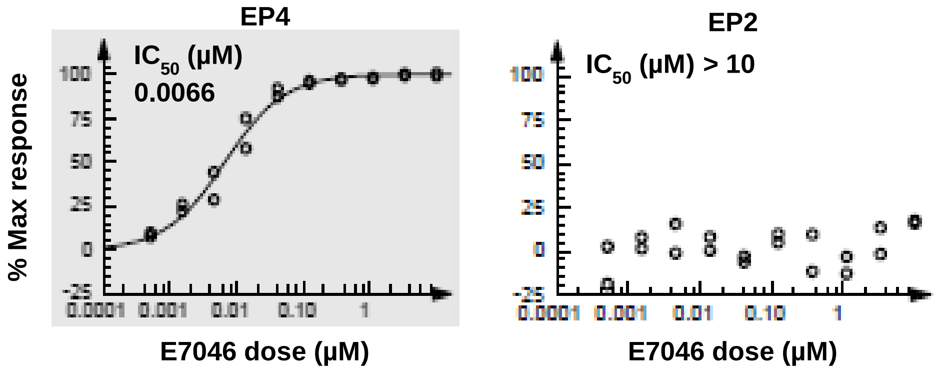

Supplementary Figure 1. Selectivity and EP4 antagonism activity of E7046. Representative dose-response graphs of E7046 antagonism of PGE_2 activity in cells expressing EP4 receptor (left graph) or EP2 receptor (right graph) were used to calculate IC_{50} values (n=2). IC50 values are indicated. Note that no significant activity of E7046 on EP2 was observed at the tested top concentration 10 µM.

A

System	Cell Type/Stimuli	Readout Parameters
LPS	Peripheral Blood Mononuclear Cells + Endothelial cells/TLR4	MCP-1,VCAM-1,TM,TF,CD40,E-selection,CD69,IL-8,IL1α,M-CSF,PGE2,SRB,TNFα,sIL-6
Sag	Peripheral Blood Mononuclear Cells + Endothelial cells/TCR	MCP-1,CD38, CD40,E-selection,CD69,IL-8,MIG,PBMC,Cytotoxicity,Proliferation,SRB,sIL-6
M1Mphg	Venular endothelial cells +M1 macrophages/TLR2 agonist	MCP-1,MIP-1α,VCAM-1,CD40,E-selection,CD69,IL-8,IL-1α,M-CSF,sIL-10,SRB,SRB-Mphg,sIL-6,VEGF,TGFβ,IL-1β,TNFα,CD206,CD163,HLA-DR
M2Mphg	Venular endothelial cells +M2 macrophages/ IL-4	MCP-1,MIP-1α,VCAM-1,CD40,E-selection,CD69,IL-8,IL-1α,M-CSF,sIL-10,SRB,SRB-Mphg,sIL-6,VEGF,TGFβ,IL-1β,TNFα,CD206,CD163,HLA-DR

B

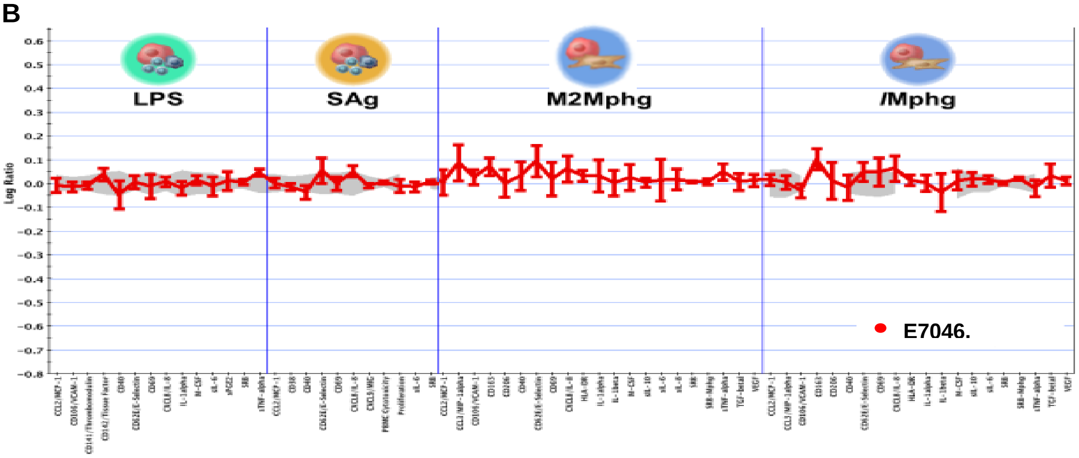

C

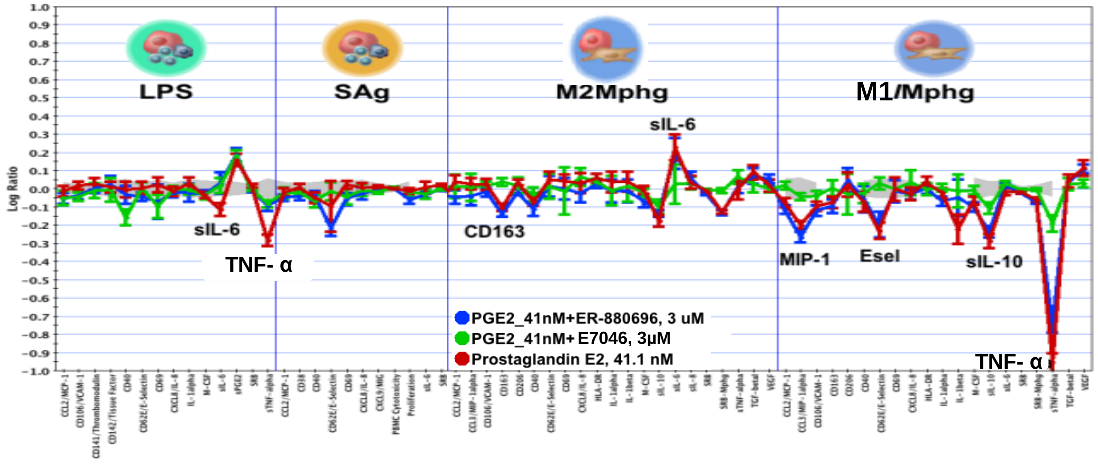

Supplementary Figure 2. E7046 reversed PGE_2-derived effects on activated human macrophages in co-culture systems. A. Table summarizing the various *in vitro* co-culture assay systems and readouts. B. In the absence of PGE_2, E7046 does not change the baseline readings for the parameters in co-culture *in vitro* assay systems. Grey line represents baseline levels of secreted molecules in the absence of PGE_2 or EP receptors antagonists. C. Activity profiles of PGE_2 + EP2 antagonist ER-880696, or E7046 generated using cellular co-culture systems described in A. Values represent mean ± SD from 3 experiments.

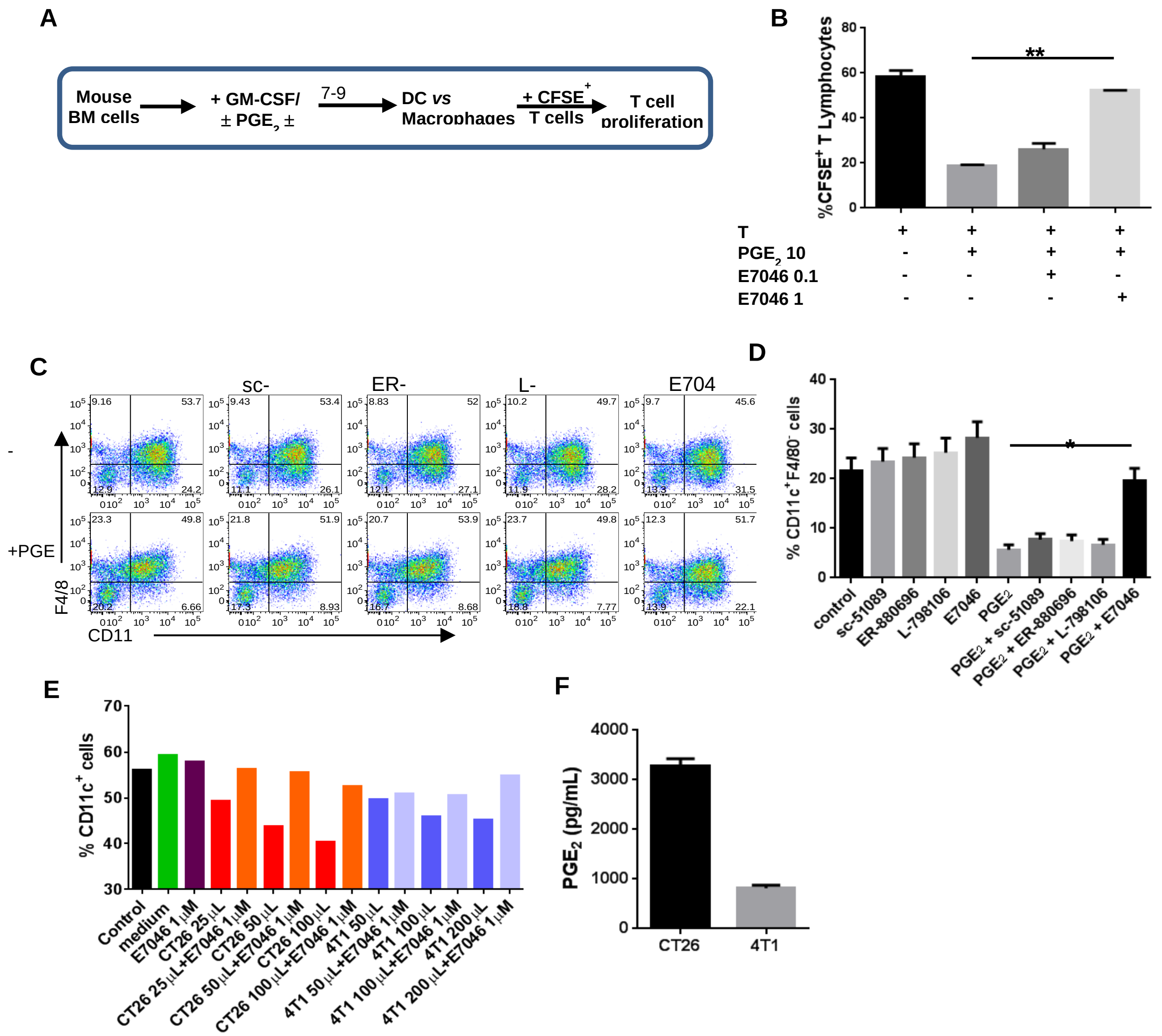

Supplementary Figure 3. Immunomodulatory effects of E7046 on mouse myeloid cells. A. Schematic representation of the assay. B. Frequencies of proliferating mouse T cells from the assay in A. Data represent mean ± SEM of duplicate samples, ***P < 0.001. C. Selective blockade of EP4 signaling in BM cells promoted DCs formation. Representative FACS dot plots showing frequencies of CD11c^{+} DCs (lower right quadrant) vs. F4/80^{+} macrophages (upper left quadrant) in the absence of PGE_2 (upper panel) or presence of PGE_2 (lower panel). EP1 antagonist sc-51089, EP2 antagonist ER-880696, EP3 antagonist L-798106, or E7046 were all added at 1 µM to the differentiating BM cells in the presence of PGE_2 (10 nM). D. DCs (CD11c^{+}F4/80^{-}) frequencies in C were plotted by treatment. Data represent mean ± SEM from two independent experiments, **P < 0.01. E. Dose-dependent inhibition of CD11c^{+} DC formation from mouse BM cells by culture medium from PGE_2-producing CT26 or 4T1 mouse cancer cell lines, with or without E7046. F. PGE_2 levels were measured in CT26 and 4T1 cell line supernatants by ELISA.

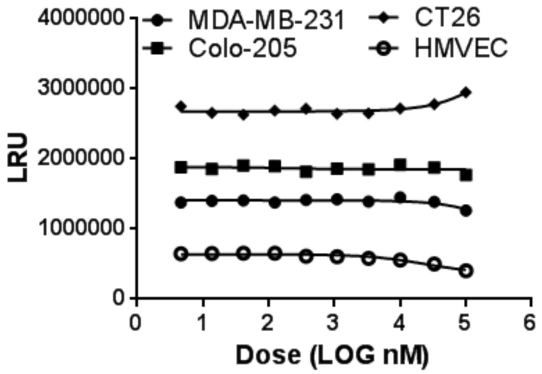

Supplementary Figure 4. Negligible effect of E7046 on the survival and proliferation of human cancer cell lines and primary endothelial cells. Representative result (n=2) showing the activity of E7046 in an *in vitro* cell growth inhibition assay on mouse (CT26), human (MDA-MB-231, Colo-205) cancer cell lines, and human microvascular endothelial cells (HMVEC).

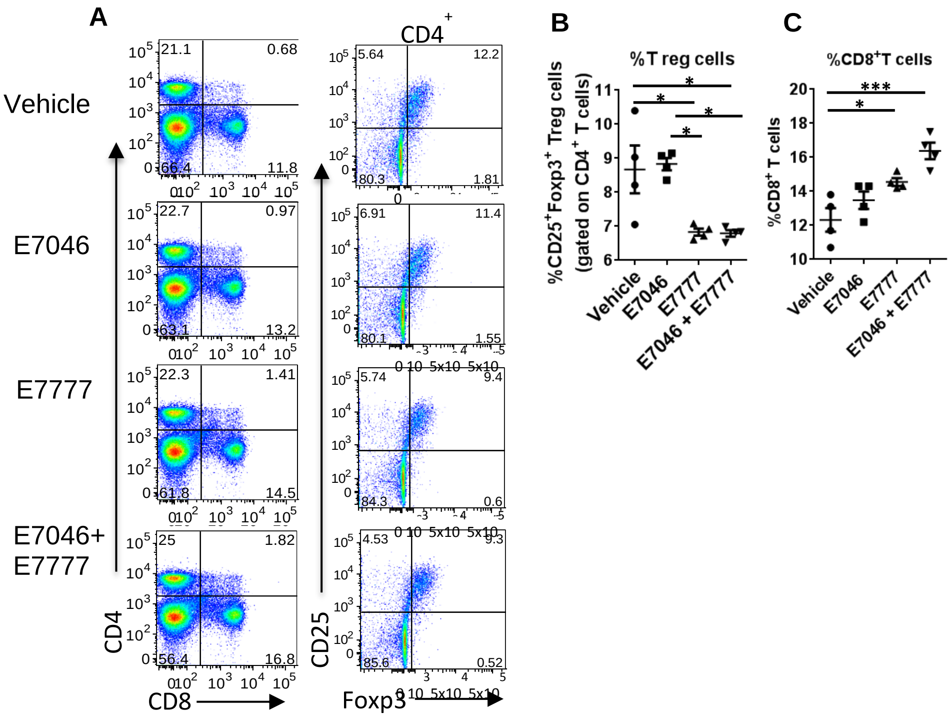

Supplementary Figure 5. E7046 + E7777 combination induced Treg cell apoptosis without affecting CD8$^+$ T cells in spleen. A. Representative FACS dot plots showing splenic CD4$^+$ and CD8$^+$ T cell frequencies (left panel) and Treg cell frequencies among CD4$^+$T cells (right panel). B. Quantification of data in A as mean ± SEM of n=4, * *P* < 0.05; ***, *P* < 0.001; One-way ANOVA.

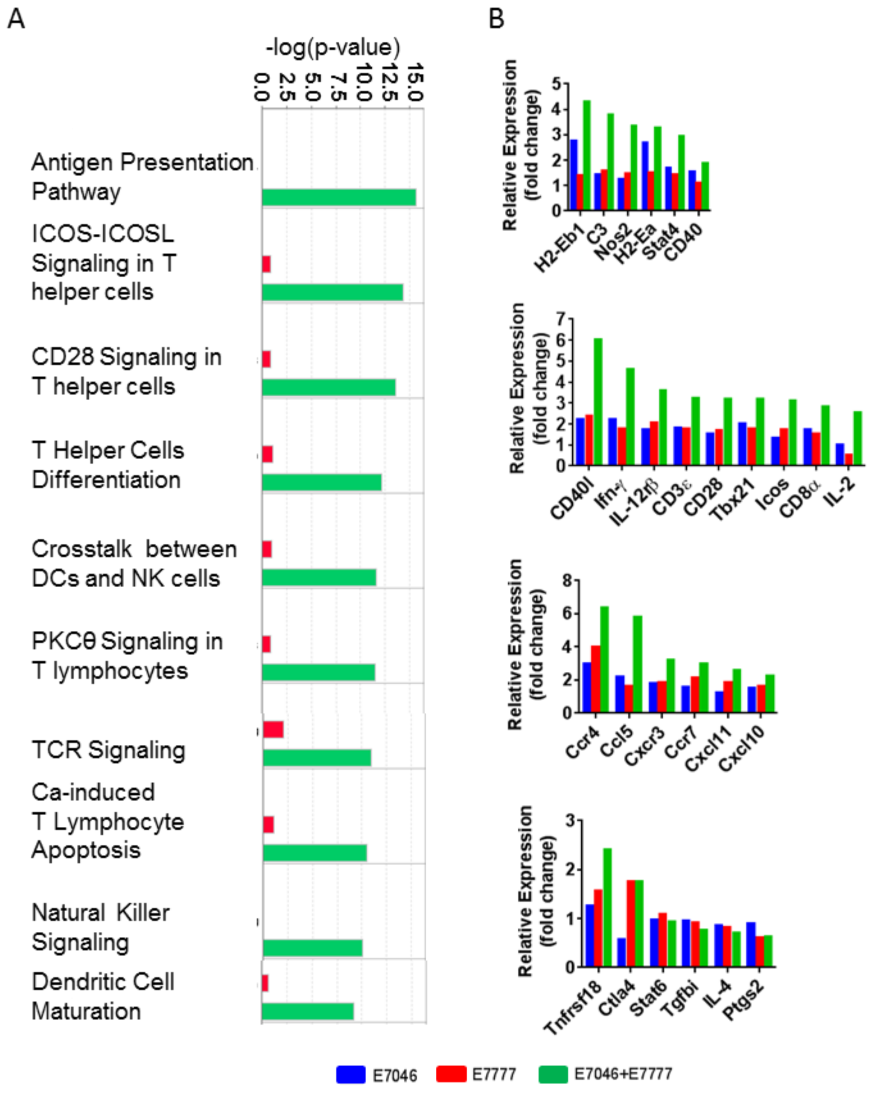

Supplementary Figure 6. E7046 + E7777 combination induced an immune gene profile consistent with acute inflammation and tumor rejection. A. Table depicting major pathways determined to be activated as a result of treatment, based on the expression of one or more genes in a pathway, determined by Ingenuity Pathway analysis. B. TLDA mRNA expression of genes involved in (from upper to lower) antigen processing & presentation/Innate Immunity, T cell activation/ Th1 differentiation/cytotoxicity, lymphocyte recruitment, and Immunosuppressive/inhibitory molecules determined in CT26 tumors isolated from mice treated with E7046, E7777, or E7046 + E7777. Expression data is shown as mean normalized fold change compared with vehicle (n=6). E7046 150 mg/kg was administered p.o. daily for 20 days, and E7777 was injected i.v. as 3 doses of 125 µg/kg every 7 days.
